# Supplementary material for: Protein-responsive protein release of supramolecular/polymer hydrogel composite integrating enzyme activation systems
Source: Nat Commun. 2020 Jul 31;11:3859. doi: 10.1038/s41467-020-17698-0 (PMC7395795; doi:10.1038/s41467-020-17698-0)
Supplement: Supplementary file 1 — Supplementary Information File [file 41467_2020_17698_MOESM1_ESM.pdf]

Supplementary information

Protein-responsive protein-release of supramolecular/polymer hydrogel composite integrating enzyme activation systems

Shigemitsu et al.

Supplementary Methods

General. Unless stated otherwise, all commercial reagents were used as received. Thin layer chromatography (TLC) was performed on silica gel $60F_{254}$ (Merck). Column chromatography was performed on silica gel 60N (Kanto Chemical, 40–50 μm). ^{1}H, ^{13}C, and ^{19}F NMR spectra were obtained on a Varian Mercury 400, a JEOL JNM-ECZ500, and a JMM-ECA600 spectrometer with tetramethylsilane for $CDCl_3$ or residual non-deuterated solvents (DMSO-d_6: 2.50 ppm for ^{1}H, 39.52 ppm for ^{13}C) as the internal references, and sodium trifluoromethanesulfonate (–78.8 ppm) for ^{19}F NMR as the external reference. ESI mass spectra were recorded using an Exactive (Thermo Scientific). Reversed-phase HPLC (RP-HPLC) was carried out on a Hitachi Chromaster system equipped with a diode array and YMC-Pack Triart C18 or ODS-A columns. All runs used linear gradients of acetonitrile (ACN) containing 0.1% trifluoroacetic acid (TFA) and 0.1 % aqueous TFA. **EAT(avidin)** were synthesized as reported.[1] DHFR were expressed as previously described.[2] UV-vis absorption spectra were recorded using a Shimadzu UV-2600 spectrometer. CD spectra were measured using a JASCO J-1500WI spectrometer. The images of confocal laser scanning microscopy (CLSM) were acquired by a LSM 800 equipped with an Airyscan unit (Carl Zeiss Microscopy). Plan-Apochromat objectives (63×, 1.40 numerical aperture, oil, for high-resolution CLSM imaging, Zeiss) were used. The Pearson's correlation coefficients were calculated by Coloc 2 program in Fiji.[3] Rheological measurements were carried out using MCR-502 (Anton Paar).

bCAII response on the hydrogel array chip. A suspension of an **APmoc-F(CF_3)F** or **Bz-FF** powder in 100 mM HEPES, pH 8.0 was heated by a heating gun (PJ-206A1, Ishizaki) until dissolving. After cooling to rt, 20 μL of the resultant solution was added to a glass plate and incubated for 2 h at 25 ºC in a humid container to avoid dryness. 6 h after addition of bCAII solution (Sigma-Aldrich, C2522, 100 μM, 2.0 μL) with/without EZA (0 or 1.0 mM), the samples were touched with a paper (prowipe S220, elleair) to judge whether the samples were gel or sol. Paper absorb sol samples, but not gel samples. The assay conditions were referred in the figure captions.

Protein response on the hydrogel array chip. A suspension of an **APmoc-F(CF_3)F** powder in 100 mM HEPES, pH 8.0 was heated by a heating gun until dissolving. After

cooling to rt, 18.0 μL of **APmoc-F(CF_3)F** solution was added to a glass plate. A couple of minutes after addition on a glass chip (sample temperature was *ca.* 30 ºC), the mixture of bCAII and EAT (2.0 μL) was added before gelation. After 2 h, protein solutions (avidin (neutralized): Wako Pure Chemical, 015-24231, 200 μM, 2.0 μL; DHFR: 450 μM, 2.0 μL; anti-DNP IgG: Thermo Fisher Scientific, 04-8300, 150 μM, 2.0 μL) were added. After incubation for 6, 12, or 18 h at 25 ºC in a humid container to avoid dryness, the samples were touched with a paper (prowipe S220, elleair) to judge whether the samples were gel or sol. Paper absorb sol samples, but not gel samples. The assay conditions were referred in the figure captions.

HPLC analysis of the hydrogel droplets. 6 h after addition of bCAII or avidin, the samples were diluted by a mixture of 1:1 CH_3CN/H_2O (60 μL) and a DMSO solution of terephthalic acid (100 mM, 2 μL). The resultant mixture was filtered with membrane filter (diameter: 0.45 μm), and then analyzed by RP-HPLC (column: YMC-Triart C18, A:B = 10:90 to 80:20 for 40 min, A: CH_3CN containing 0.1% TFA, B: H_2O containing 0.1% TFA).

CLSM imaging of the APmoc-F(CF_3)F hydrogel. The suspension of **APmoc-F(CF_3)F** in 100 mM HEPES, pH 8.0 (0.35 wt%) was heated by a heating gun until dissolving. After cooling to rt, the resultant mixture (18 μL) was transferred to a glass bottom dish (Matsunami). A couple of minutes after addition on a glass chip (sample temperature was *ca.* 30 ºC), the mixture of bCAII and **EAT(avidin)** (100 μM, 200 μM, respectively, 2 μL in 100 mM HEPES, pH 8.0) was added and incubated for 15 min at rt. To the resultant hydrogel, a solution of avidin (200 μM, 2 μL), buffer, or avidin premixed with biotin (200 μM, 1.2 mM, respectively, 2 μL) was added. After incubation for 6 h, a solution of **TMR-Gua** (280 μM, 1 μL, 1:9 DMSO/100 mM HEPES (pH 8.0)) was added, and CLSM imaging was subsequently conducted.

Preparation of the composite hydrogel containing bCAII/EAT. A suspension of an agarose powder in 100 mM HEPES, pH 8.0 was heated for 5 min by heating until dissolving. The hot agarose solution was added to an **APmoc-F(CF_3)F** powder, and the mixture was heated by a heating gun until dissolving. The resultant mixture (200 μL) was transferred to a vial or a PDMS mold (pore diameter: 10 mm). Before gelation, a

mixture of bCAII and EAT (20 μL) was added. After incubation for 2 h in a humid container to avoid dryness, the resultant composite hydrogel was used for response tests and rheological experiments.

CLSM imaging of the composite hydrogel. A suspension of a FL-agarose powder in 100 mM HEPES, pH 8.0 was heated for 5 min by a heating gun until dissolving. The hot FL-agarose solution was added to an **APmoc-F(CF_3)F** powder. The resultant mixture was heated again by a heating gun until dissolving. Before gelation, the mixture (20 μL) was transferred to a glass bottom dish (Matsunami). After incubation for 15 min at rt, a solution of **TMR-Gua** (280 μM, 1 μL, 1:9 DMSO/100 mM HEPES (pH 8.0)) was added to the composite hydrogel, and then CLSM imaging was carried out. The detailed assay conditions were referred in the figure captions.

HPLC analysis of the composite hydrogel. The stock solution of avidin (200 μM, 20 μL) was added to the composite hydrogel. After incubation at rt for 16 h, DMF (600 μL) and a DMSO solution of terephthalic acid (100 mM, 30 μL) were added to the composite hydrogel, and the resultant mixture was dissolved by vortex mixing. The mixture was filtered and then analyzed by RP-HPLC (column: YMC-Triart C18, A:B = 10:90 to 80:20 for 40 min, A: CH_3CN containing 0.1% TFA, B: H_2O containing 0.1% TFA).

CD spectroscopy. The sample was poured into a quartz cell before gelation (optical length: 0.05 mm). After incubation for 10 min at room temperature, CD spectra were measured.

Scanning electron microscopy. APmoc-F(CF_3)F, agarose, and composite hydrogels were frozen by immersing in liquid nitrogen and lyophilized overnight. The samples were put on a conductive carbon adhesive tape (thin aluminum foil core) and sputter-coated with a thin layer of platinum (*ca.* 5 nm). The secondary electron images were acquired by a field emission scanning electron microscope (Hitachi, SU8200) at a 1.5 kV voltage.

Protein release experiments. Proteins were embedded in the composite hydrogel (20 μL) by addition of protein solutions before gelation (sample temperature was *ca.* 30 ºC). After moving from the PDMS mold to a vial, the solution of avidin (200 μM, 2 μL) was added to the composite hydrogel. After incubation for 16 (for avidin) or 24 h (for RNase A) at rt in a humid container to avoid dryness, a HEPES buffer (100 mM, pH 8.0, 20 μL) was added to the resultant gel, and subsequently incubated for 3 h at rt. 10 μL of supernatant was picked out, and mixed with a Laemmli buffer (5-times higher concentration containing 10 vol% 2-mercaptoethanol and 3 mM biotin). The resultant mixture was heated at 95 ºC for 5 min, analyzed by SDS-PAGE, and quantified by ChemiDoc-XRS (observed at 595 nm).

Rheological analysis. The resultant disk-shaped composite hydrogels (*ca.* 10 mm) were carefully took out from the PDMS mold and put onto the stage of a rheometer (MCR-502, Anton Paar) with a parallel plate geometry. Strain sweep data were obtained using shear mode at a frequency of 10 rad/s, and linear dynamic viscoelasticity were measured in shear mode at 1% strain amplitude for frequency sweep.

UV-Vis absorption spectroscopy of FL-agarose. FL-agarose (0.50 mg) was suspended in 10 mM tetraborate buffer (500 μL). The suspension was heated until dissolving with a heating gun for 5 min (concentration of repeating units: 3.26 mM). The resultant solution was measured by a UV-Vis spectrometer to determine absorbance derived from fluorescein to be 0.0365 (0.487 μM, molar absorption coefficient: 75,000[71]), corresponding to 0.015 mol% relative to the repeating unit of agarose (supplementary Fig. 40).

Modification of myoglobin with Alexa fluor 647. To a PBS solution of myoglobin (Sigma-Aldrich, M1882, 0.5 mg/mL, 6 mL, pH 8.0) was added a DMSO solution of Alexa fluor 647-NHS ester (Thermo Fisher Scientific, A-20006, 50 mM, 6.72 μL). The reaction mixture was incubated at 4 ºC for 24 h. The resulting mixture was diluted with PBS (18 mL) and dialyzed by Spectra/Por dialysis membrane (MWCO 8000) against PBS (500 mL, 3 times) and 100 mM HEPES (1 L, pH 8.0). The solution was concentrated by an Amicon-Ultra Centrifugal filter unit (NMWL 3500) to obtain Ax647-Mb as a blue transparent solution (Mb: 718 μM, Alexa fluor 647: 775 μM

determined by UV-vis absorption spectroscopy in supplementary Fig. 41). The molar absorption coefficients of Mb (18,800[72]) and Alexa Flour 647 (290,000[71]) were used.

Determination of the activity of RNase A. The enzymatic activity of RNase A was monitored by DNase+RNase detection kit (Jena Bioscience). The supernatant solution was diluted by 1000-fold with a detection buffer. The resultant solution (10 µL) and the master mix containing a probe (40 µL) were mixed on ice. The time course of fluorescent intensity was monitored by a plate reader (infinite M200, TECAN, excitation wavelength: 495 nm, emission wavelength: 520 nm, gain: 100, interval: 1 min, temperature: 37 ºC).

Acquisition condition of Airyscan CLSM imaging

*2D imaging of **APmoc-F(CF$_3$)F** hydrogel (Fig. 2e)*

Image size: 71.63 µm × 71.63 µm, 2136 pixel × 2136 pixel

	TMR channel
Laser wavelength (nm)	561
Laser power (%)	0.10
Scan mode	Frame
Scan zoom	X: 1.4, Y: 1.4
Pixel time (µs)	31.28
Scan direction	Unidirectional
Averaging	1
Detection wavelength (nm)	450–700
Detector gain (V)	850
Detector offset	0
Detector digital gain	1.0
Airyscan mode	2D SR, Manual, 4.0

2D imaging of composite hydrogel (Fig. 4a)

Image size: 33.00 µm × 33.00 µm, 984 × 984 pixel

	TMR channel	FL channel
Laser wavelength (nm)	561	488
Laser power (%)	0.30	0.20
Scan mode	Frame	Frame
Scan zoom	X: 3.0, Y: 3.0	X: 3.0, Y: 3.0
Pixel time (µs)	67.06	67.06
Scan direction	Unidirectional	Unidirectional
Averaging	1	1
Detection wavelength (nm)	450–700	450–545
Detector gain (V)	850	850
Detector offset	0	0
Detector digital gain	1.0	1.0
Airyscan mode	2D SR, Manual, 5.0	2D SR, Manual, 4.0

3D imaging of composite hydrogel (Fig. 4c)

Z-Stack: 11 slices (1.8 µm)

Image size: 33.80 µm × 33.80 µm, 1008 pixel × 1008 pixel

	TMR channel	FL channel
Laser wavelength (nm)	561	488
Laser power (%)	0.30	0.05
Scan mode	Frame	Frame
Scan zoom	X: 3.0, Y: 3.0	X: 3.0, Y: 3.0
Pixel time (µs)	8.38	8.38
Scan direction	Unidirectional	Unidirectional
Averaging	2	2
Detection wavelength (nm)	450–700	450–545
Detector gain (V)	850	900
Detector offset	0	0
Detector digital gain	1.0	1.0
Airyscan mode	2D SR, Manual, 5.0	2D SR, Manual, 4.0

2D imaging of composite hydrogel with Ax647-Mb (Fig. 5c)

Image size: 71.69µm × 71.69 µm, 2304 pixel × 2304 pixel

	Alexa647 channel	TMR channel	FL channel
Laser wavelength (nm)	640	561	488
Laser power (%)	0.10	0.10	0.40
Scan mode	Frame	Frame	Frame
Scan zoom	X: 1.4, Y: 1.4	X: 1.4, Y: 1.4	X: 1.4, Y: 1.4
Pixel time (µs)	14.50	14.50	14.50
Scan direction	Unidirectional	Unidirectional	Unidirectional
Averaging	1	1	1
Detection wavelength (nm)	631–700	528–594	450–544
Detector gain (V)	930	901	850
Detector offset	0	0	0
Detector digital gain	1.0	1.0	1.0
Airyscan mode	2D SR, Manual, 4.0	2D SR, Manual, 4.0	2D SR, Manual, 4.0

Organic syntheses

APmoc-FF

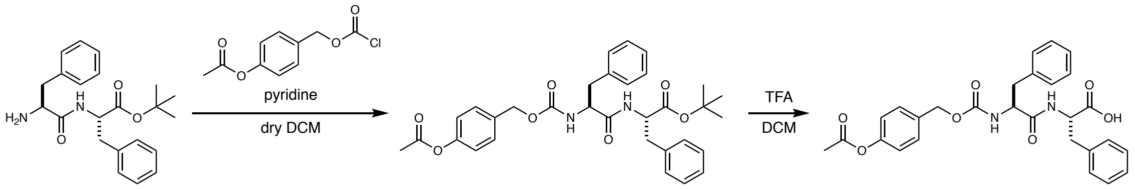

To a solution of **H-FF-OtBu** (80.7 mg, 219 μmol, 1.0 eq) and 4-(((chlorocarbonyl)oxy) methyl)phenyl acetate (50.0 mg, 219 μmol, 1.0 eq) in dry DCM (5 mL) were added pyridine (176 μL, 2.19 mmol, 10 eq). The mixture was stirred at room temperature (rt) for 2 h under N_2 atmosphere. After completion of the reaction, the solvent was removed under reduced pressure. The reactant was diluted with DCM (30 mL), and washed with 5 % citric acid (30 mL×3), sat. $NaHCO_3$ (30 mL×3), H_2O (30 mL) and brine (30 mL). The organic layer was dried over Na_2SO_4 and concentrated *in vacuo* to afford **APmoc-FF-OtBu** (86.3 mg, 154 μmol, 70 %) as a white solid.

APmoc-FF-OtBu was dissolved in DCM (4 mL) and TFA (2 mL). The mixture was stirred at rt for 2 h. After completion of the reaction, the solvent was removed under reduced pressure. The crude product was purified by RP-HPLC (column: YMC ODS-A, solvent gradient: A:B = 20:80 to 80:20 for 10 min, A: CH_3CN with 0.1% TFA, B: H_2O with 0.1% TFA) to give **APmoc-FF** as a white solid (35.4 mg, 70.2 μmol, 46 %).

^{1}H NMR (500 MHz, DMSO-d_6, rt): δ 12.7 (br, 1H), 8.28 (d, J = 7.5 Hz, 2H), 7.47 (d, J = 9.0 Hz, 1H), 7.29–7.17 (m, 12H), 7.08 (d, J = 9.0 Hz, 2H), 4.92 (s, 2H), 4.48–4.44 (m, 1H), 4.29–4.24 (m, 1H), 3.10–3.06 (m, 1H), 2.97–2.89 (m, 2H), 2.70–2.65 (m, 1H), 2.26 (s, 3H).

^{13}C NMR (125 MHz, DMSO-d_6, rt): δ 172.8, 171.53, 169.2, 155.7, 149.9, 138.0, 137.4, 134.5, 129.2*, 128.6, 128.2, 128.0, 126.5, 126.2, 121.7, 64.6, 56.0, 53.5, 37.4, 36.7, 20.8.

HR-FTMS (ESI): Calcd. For $[M+Na]^+$: m/z = 527.1789; found: 527.1790

*:two peaks were overlapped.

APmoc-F(CF_3)F

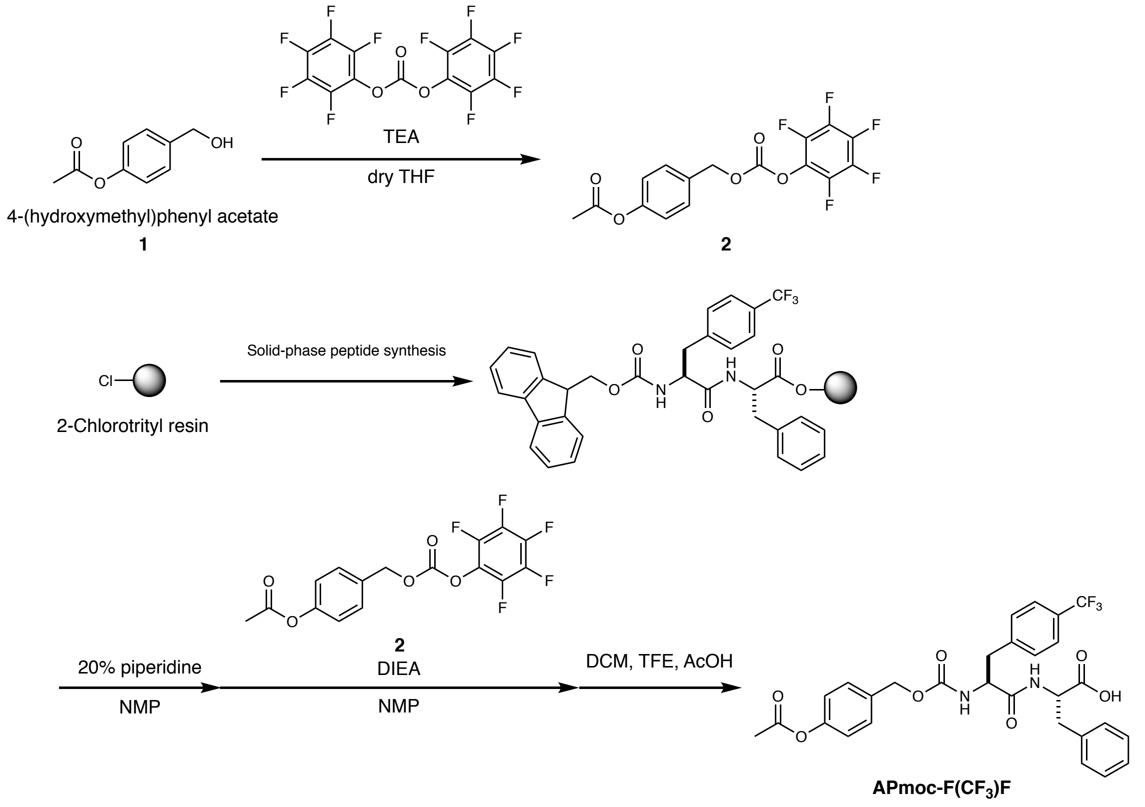

Compound 2

To a solution of 4-(hydroxymethyl)phenyl acetate **1** (784 mg, 4.72 mmol, 1.0 eq) in dry THF (10 mL) were added bis(pentafluorophenyl) carbonate (2.79 g, 7.08 mmol, 1.5 eq) and triethylamine (TEA) (1.96 mL, 14.1 mmol, 3.0 eq) at 0 ºC. The mixture was stirred at 0 ºC for 2.5 h under N_2 atmosphere. After completion of the reaction, the solvent was removed under reduced pressure. The crude product was purified by silicagel column chromatography (eluent: $CHCl_3$) to afford **2** (1.92 g, quant.) as a white solid.

^{1}H NMR (400 MHz, $CDCl_3$, rt): δ 7.45 (d, J = 8.4 Hz, 2H), 7.15 (d, J = 8.4 Hz, 2H), 5.32 (s, 2H), 2.32 (s, 3H).

^{13}C NMR (125 MHz, DMSO-d_6, rt): δ 169.6, 151.3, 141.3 (ddt, J_{CF} = 252.7, 12.7, 4.2 Hz), 139.9 Hz (dtt, J_{CF} = 236.8, 13.8 Hz, J_{CF} = 4.2 Hz), 137.9 (dtd, J_{CF} = 245.3, 13.3, 3.1 Hz), 131.4, 129.9, 122.09, 125.7 (tt, J_{CF} =11.6, 1.6 Hz), 71.4, 21.1.

^{19}F NMR (470 MHz, DMSO-d_6, rt): δ –153.51 (d, J = 17.4 Hz, 2F), –157.86 (t, J = 21.6 Hz, 1F), –162.46 (d, J = 20.2 Hz, 2F).

HR-FTMS (ESI): Calcd. For $[M+Na]^+$: m/z = 399.0262; found: 399.0264

APmoc-F(CF_3)F

APmoc-F(CF_3)F was prepared by Fmoc solid-phase peptide synthesis using a commercially available 2-chlorotrityl resin (148 mg, 241 μmol, 1.0 eq). The condensation reaction was carried out in the presence of Fmoc-protected amino acids (3.0 eq), 2-(1*H*-benzotriazol-1-yl)-1,1,3,3-tetramethyluronium hexafluorophosphate (HBTU: 3.0 eq), 1-hydroxybenzotriazole hydrate (HOBt·H_2O: 3.0 eq) and diisopropylethylamine (DIEA: 6.0 eq) in 1-methyl-2-pyrrolidinone (NMP). Removal of Fmoc protecting group was performed using NMP solution containing 20% piperidine. After removal of the Fmoc group on the terminal amino group, the resulting free amino group was allowed to react with **2** (3.0 eq) in the presence of DIEA (6.0 eq) in NMP. Finally, cleavage of the compound from the resin were performed using a cocktail (7:2:1 CH_2Cl_2/trifluoroethanol (TFE)/AcOH) at rt for 1.5 h to give crude product of **APmoc-F(CF_3)F** as a colorless oil. The crude product was purified by RP-HPLC (column: YMC ODS-A, solvent gradient: A:B = 20:80 to 80:20 for 40 min, A: CH_3CN with 0.1% TFA, B: H_2O with 0.1% TFA) to give **APmoc-F(CF_3)F** as a white solid (29.0 mg, 50.7 μmol, 21%).

^{1}H NMR (600 MHz, DMSO-d_6, rt): δ 8.34 (d, J = 7.8 Hz, 1H), 7.62 (d, J = 7.8 Hz, 2H), 7.55 (d, J = 9.6 Hz, 1H), 7.48 (d, J = 7.8 Hz, 2H), 7.29–7.20 (m, 7H), 7.06 (d, J = 7.2 Hz, 2H), 4.92 (s, 2H), 4.49–4.46 (m, 1H), 4.34–4.30 (m, 1H), 3.09-3.03 (m, 2H), 2.97–2.94 (m, 1H), 2.80–2.75 (m, 1H), 2.26 (s, 3H).

^{13}C NMR (150 MHz, DMSO-d_6, rt): δ 172.7, 171.2, 169.1, 155.7, 149.9, 143.0, 137.3, 134.5, 130.0, 129.2, 128.6, 128.2, 127.0 (q, J_{CF} = 31.8 Hz), 126.5, 124.8, 124.4 (q, J_{CF} = 272.1 Hz), 121.6, 64.7, 55.5, 53.5, 37.2, 36.6, 20.8.

^{19}F NMR (470 MHz, DMSO-d_6, rt): δ –61.3 (s, 3F).

HR-FTMS (ESI): Calcd. For $[M+Na]^+$: m/z = 595.1663; found: 595.1667

APmoc-F(F)F

APmoc-F(F)F was prepared from 2-chlorotrityl resin (151 mg, 246 μmol) in the same way for **APmoc-F(CF_3)F** and was obtained in 35% (45.4 mg, 86.9 μmol) as a white solid.

^{1}H NMR (500 MHz, DMSO-d_6, rt): δ 12.7 (brs, 1H), 8.28 (d, J = 7.5 Hz, 2H), 7.47 (d, J = 9.0 Hz, 1H), 7.30–7.19 (m, 9H), 7.08–7.03 (m, 4H), 4.95–4.89 (m, 2H), 4.48–4.45 (m,

1H), 4.27–4.22 (m, 1H), 3.10–3.06 (m, 1H), 2.96–2.90 (m, 2H), 2.69–2.64 (m, 1H), 2.26 (s, 3H).

^{13}C NMR (125 MHz, DMSO-d_6, rt): δ 172.7, 171.4, 169.2, 160.9 (d, J_{CF} = 242.1 Hz), 155.7, 149.9, 137.4, 134.5, 134.1 (d, J_{CF} = 2.7 Hz), 131.0 (d, J_{CF} = 7.4 Hz), 129.2, 128.6, 128.2, 126.5, 121.7, 114.7 (d, J_{CF} = 21.2 Hz), 64.6, 56.0, 53.5, 36.7, 36.6, 20.8.

^{19}F NMR (470 MHz, DMSO-d_6, rt): δ –117.5 (tt, J_{HF} = 9.4, 5.4 Hz, 1F).

HR-FTMS (ESI): Calcd. For $[M+Na]^+$: *m/z* = 545.1695; found: 545.1696

APmoc-FF(F)

APmoc-FF(F) was prepared from 2-chlorotrityl resin (151 mg, 246 μmol) in the same way for **APmoc-F(CF$_3$)F** and was obtained in 17% (21.3 mg, 40.8 μmol) as a white solid.

^{1}H NMR (500 MHz, DMSO-d_6, rt): δ 12.8 (brs, 1H), 8.27 (d, *J* = 7.5 Hz, 2H), 7.47 (d, *J* = 9.0 Hz, 1H), 7.29–7.23 (m, 9H), 7.10–7.05 (m, 4H), 4.92 (S, 2H), 4.46–4.42 (m, 1H), 4.28–4.23 (m, 1H), 3.09–3.05 (m, 1H), 2.96–2.91 (m, 2H), 2.69–2.64 (m, 1H), 2.26 (s, 3H).

^{13}C NMR (125 MHz, DMSO-d_6, rt): δ 172.6, 171.5, 169.2, 161.1 (d, J_{CF} = 241.8 Hz), 155.7, 149.9, 138.0, 134.5, 133.5 (d, J_{CF} = 2.7 Hz), 131.1 (d, J_{CF} = 7.7 Hz), 129.2, 128.6, 128.2, 126.2, 121.7, 114.8 (d, J_{CF} = 21.2 Hz), 64.6, 56.0, 53.5, 37.4, 35.8, 20.8.

^{19}F NMR (470 MHz, DMSO-d_6, rt): δ –117.3 (m, 1F).

HR-FTMS (ESI): Calcd. For $[M+Na]^+$: *m/z* = 545.1695; found: 545.1696

APmoc-F(F)F(F)

APmoc-F(F)F(F) was prepared from 2-chlorotrityl resin (151 mg, 246 μmol) in the same way for **APmoc-F(CF$_3$)F** and was obtained in 23% (31.6 mg, 58.8 μmol) as a white solid.

^{1}H NMR (500 MHz, DMSO-d_6, rt): δ 12.8 (brs, 1H), 8.29 (d, *J* = 8.0 Hz, 2H), 7.48 (d, *J* = 9.0 Hz, 1H), 7.30–7.25 (m, 6H), 7.10–7.03 (m, 6H), 4.93 (m, 2H), 4.47–4.42 (m, 1H), 4.26–4.21 (m, 1H), 3.09–3.05 (m, 1H), 2.95–2.91 (m, 2H), 2.69–2.64 (m, 1H), 2.26 (s, 3H).

^{13}C NMR (125 MHz, DMSO-d_6, rt): δ 172.6, 171.4, 169.2, 161.1 (d, J_{CF} = 242.7 Hz), 160.9 (d, J_{CF} = 241.9 Hz), 155.7, 149.9, 134.5, 134.1 (d, J_{CF} = 2.7 Hz), 133.5 (d, J_{CF} =

3.2 Hz), 131.0 (d, J_{CF} = 7.7 Hz), 131.0 (d, J_{CF} = 7.7 Hz), 128.6, 121.7, 114.8 (d, J_{CF} = 20.7 Hz), 114.7 (d, J_{CF} = 20.1 Hz), 64.6, 56.0, 53.5, 36.6, 35.8, 20.8.

^{19}F NMR (470 MHz, DMSO-d_6, rt): δ –117.3 (tt, J_{HF} = 8.7, 5.6 Hz, 1F), –117.5 (tt, J_{HF} = 9.2, 5.6 Hz, 1F).

HR-FTMS (ESI): Calcd. For $[M+Na]^+$: *m/z* = 563.1606; found: 563.1600

APmoc-FF(CF$_3$)

APmoc-FF(CF$_3$) was prepared from 2-chlorotrityl resin (151 mg, 246 µmol) in the same way for **APmoc-F(CF$_3$)F** and was obtained in 26% (40.0 mg, 62.4 µmol) as a white solid.

^{1}H NMR (500 MHz, DMSO-d_6, rt): δ 12.8 (brs, 1H), 8.35 (d, J = 8.0 Hz, 2H), 7.63 (d, J = 8.5 Hz, 1H), 7.49–7.46 (m, 3H), 7.26–7.24 (m, 7H), 7.20–7.18 (m, 1H), 7.07 (d, J = 8.5 Hz, 6H), 4.84 (d, J = 13.0 Hz, 1H), 4.89 (d, J = 13.0 Hz), 4.54–4.50 (m, 1H), 4.28–4.23 (m, 1H), 3.21–3.17 (m, 1H), 3.06–3.02 (m, 1H), 2.95–2.90 (m, 1H), 2.69–2.63 (m, 1H), 2.26 (s, 3H).

^{13}C NMR (125 MHz, DMSO-d_6, rt): δ 172.4, 171.6, 169.2, 155.7, 149.9, 142.4, 138.0, 134.5, 130.1, 129.1, 128.6, 128.0, 127.2 (d, J_{CF} = 31.8 Hz), 126.2, 124.9 (d, J_{CF} = 3.7 Hz), 124.4 (q, J_{CF} = 271.8 Hz), 121.7, 64.6, 56.0, 53.0, 37.3, 36.4, 20.8.

^{19}F NMR (470 MHz, DMSO-d_6, rt): δ –61.3 (s, 3F).

HR-FTMS (ESI): Calcd. For $[M+Na]^+$: *m/z* = 595.1663; found: 595.1662

APmoc-F(CF$_3$)F(CF$_3$)

APmoc-F(CF$_3$)F(CF$_3$) was prepared from 2-chlorotrityl resin (151 mg, 246 µmol) in the same way for **APmoc-F(CF$_3$)F** and was obtained in 35% (45.4 mg, 73.0 µmol) as a white solid.

^{1}H NMR (500 MHz, DMSO-d_6, rt): δ 12.8 (br, 1H), 8.41 (d, J = 8.0 Hz, 2H), 7.64–7.61 (m, 4H), 7.57 (d, J = 9.0 Hz, 1H), 7.48–7.41 (m, 4H), 7.26 (d, J = 8.8 Hz, 2H), 7.06 (d, J = 8.8 Hz, 2H), 4.94 (d, J = 12.5 Hz, 1H), 4.90 (d, J = 12.5 Hz), 4.55–4.51 (m, 1H), 4.33–4.28 (m, 1H), 3.21–3.17 (m, 1H), 3.07–2.99 (m, 2H), 2.80–2.75 (m, 1H), 2.26 (s, 3H).

^{13}C NMR (125 MHz, DMSO-d_6, rt): δ 172.4, 171.2, 169.1, 155.7, 149.9, 143.0, 142.4, 134.5, 130.1, 130.0, 128.6, 127.2 (d, J_{CF} = 31.5 Hz), 127.0 (d, J_{CF} = 31.3 Hz), 124.9 (d,

J_{CF} = 4.0 Hz), 124.9 (d, J_{CF} = 3.7 Hz), 124.43 (q, J_{CF} = 271.3 Hz), 124.41 (q, J_{CF} = 271.3 Hz), 121.7, 64.7, 56.0, 53.1, 37.1, 36.4, 20.8.
^{19}F NMR (470 MHz, DMSO-d_6, rt): δ –61.3 (s, 3F), –61.4 (s, 3F).
HR-FTMS (ESI): Calcd. For $[M+Na]^+$: *m/z* = 663.1536; found: 663.1535

Bz-FF

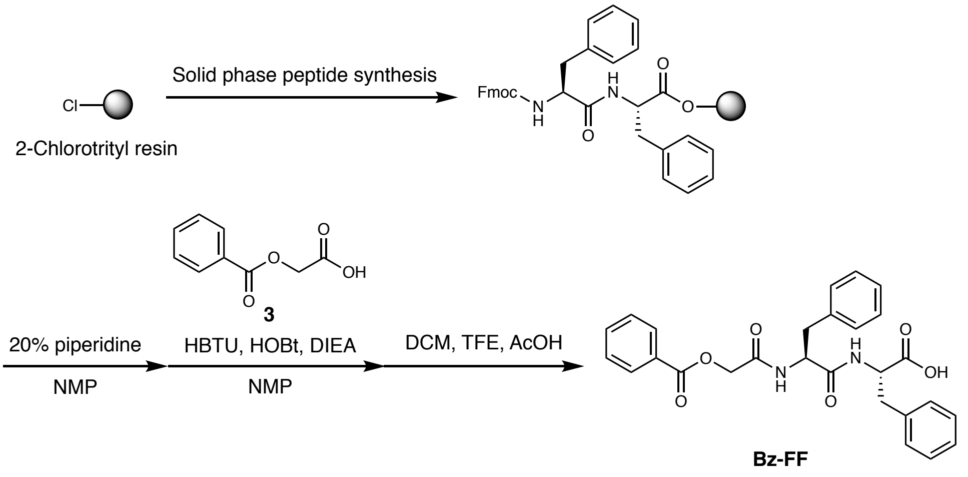

Bz-FF was prepared by Fmoc solid-phase peptide synthesis using a commercially available 2-chlorotrityl resin (140.0 mg, 210 μmol, 1.0 eq). The condensation reaction was carried out in the presence of Fmoc-protected amino acids (3.0 eq), 2-(1*H*-benzotriazol-1-yl)-1,1,3,3-tetramethyluronium hexafluorophosphate (HBTU: 3.0 eq), 1-hydroxybenzotriazole hydrate (HOBt·H_2O: 3.0 eq.) and diisopropylethylamine (DIEA: 6.0 eq) in 1-methyl-2-pyrrolidinone (NMP). Removal of Fmoc protecting group was performed using NMP solution containing 20% piperidine. After removal of the Fmoc group on the terminal amino group, the resulting free amino group was allowed to react with **3** (1.5 eq) in the presence of HBTU (1.5 eq), HOBt•H_2O (1.5 eq) and DIEA (3.0 eq) in NMP. Finally, cleavage of the compound from the resin was performed using a cocktail (7:2:1 CH_2Cl_2/TFE/AcOH) at rt for 1.0 h to give crude product of **Bz-FF** as a white solid. The crude product was purified by RP-HPLC (column: YMC ODS-A, solvent gradient: A:B = 50:50 to 70:30 for 20 min, A: CH_3CN with 0.1% TFA, B: H_2O with 0.1% TFA) to give **Bz-FF** as a white solid (38.9 mg, 82.0 μmol, 39 %).
^{1}H NMR (600 MHz, DMSO-d_6, rt): δ 8.41 (d, J = 7.2 Hz, 1H), 8.24 (d, J = 8.4 Hz, 1H), 7.97 (dd, J = 8.4, 1.2 Hz, 2H), 7.69 (t, J = 7.5 Hz, 1H), 7.55 (t, J = 7.8 Hz, 2H), 7.28–7.17 (m, 10H), 4.67 (d, J = 14.4 Hz, 1H), 4.63 (d, J =15.0 Hz, 1H), 4.61–4.57 (m, 1H),

4.47–4.44 (m, 1H), 3.08 (dd, J = 15.1, 5.1 Hz, 1H), 3.01 (dd, J = 13.8, 4.2 Hz, 1H), 2.93 (dd, J = 15.0, 9.0 Hz, 1H), 2.78 (dd, J = 13.8, 9.0 Hz, 1H).

^{13}C NMR (150 MHz, DMSO-d_6, rt): δ 172.7, 170.8, 166.2, 165.1, 137.5, 137.4, 133.5, 129.4, 129.3, 129.2, 129.1, 128.7, 128.2, 128.0, 126.4, 126.3, 62.6, 53.5, 53.3, 37.4, 36.6.

HR-FTMS (ESI): Calcd. For $[M+Na]^+$: m/z = 497.1688; found: 497.1683

EAT(avidin, long)

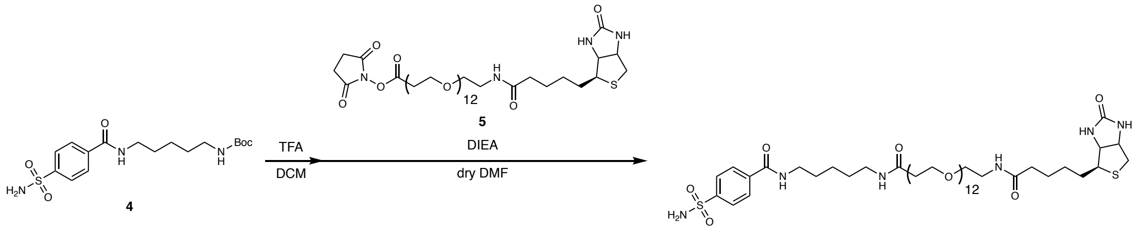

To a dry DCM solution (1 mL) of **4** (3.0 mg, 7.8 μmol, 1.0 eq) was added TFA (0.5 mL). The reaction mixture was allowed to stir at rt for 30 min. After solvent removal under reduced pressure, the resulting residue was dissolved in dry DMF (500 μL). To this solution, **5** (7.3 mg, 7.8 μmol, 1.0 eq) and DIEA (6.8 μL, 39 μmol, 5.0 eq) were added. The mixture was allowed to stir at rt for 30 min. The solvent was removed under reduced pressure. The crude product was purified by RP-HPLC (column: YMC ODS-A, solvent gradient: A:B = 20:80 to 80:20 for 40 min, A: CH_3CN with 0.1% TFA, B: H_2O with 0.1% TFA) to afford **EAT(avidin, long)** (8.0 mg, 7.2 μmol, 92%) as a colorless oil.

^{1}H NMR (400 MHz, CD_3OD, rt): δ 7.97 (s, 4H), 4.49 (dd, J = 7.6, 5.2 Hz, 1H), 4.30 (dd, J = 8.0, 4.8 Hz, 1H), 3.70 (t, J = 6.0 Hz, 2H), 3.64–3.52 (m*), 3.40 (t, J = 7.2 Hz, 2H), 3.35 (t, J = 5.6 Hz, 2H), 3.22–3.18 (m, 4H), 2.92 (dd, J = 12.8, 4.8 Hz, 1H), 2.70 (d, J = 12.8 Hz, 1H), 2.41 (t, J = 6.0 Hz, 2H), 2.22 (t, J = 6.0 Hz, 2H), 1.76–1.38 (m, 6H).

HR-FTMS (ESI): Calcd. For $[M+H]^+$: m/z = 1111.5513; found: 1111.5493

*: Peaks corresponding to ethylene glycol were overlapped with the water peak.

EAT(DHFR)

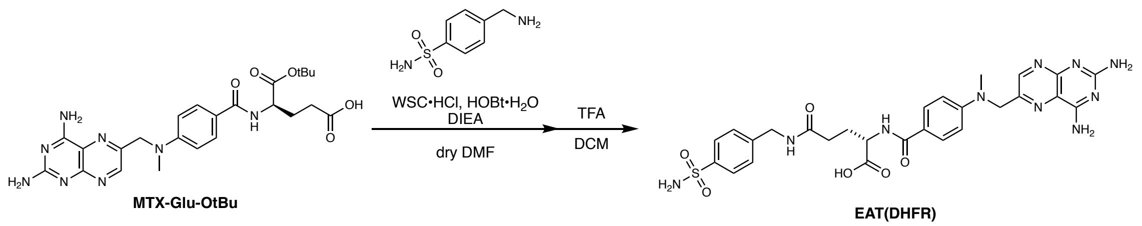

To a solution of **MTX-Glu-OtBu** (16.4 mg, 32.1 μmol, 1.0 eq), homosulfamine hydrochloride (7.2 mg, 32.3 μmol, 1.0 eq), HOBt•H_2O (5.9 mg, 38.5 μmol, 1.5 eq), WSC•HCl (10.5 mg, 54.6 μmol, 1.7 eq) in dry DMF (1.0 mL) was added DIEA (22.4 μL, 128 μmol, 4.0 eq). The reaction mixture was allowed to stir at rt for 24 h under Ar atmosphere. The solvent was removed under reduced pressure. The crude product was purified by silica gel column chromatography (eluent: $CHCl_3$ to 1:5 $CHCl_3/CH_3OH$) to obtain tBu ester-protected **EAT(DHFR)** (13.0 mg) as a yellow solid. tBu ester-protected **EAT(DHFR)** was deprotected in a 1:1 TFA/CH_2Cl_2 at rt for 2.5 h. The solvent was removed under reduced pressure. The crude product was purified by RP-HPLC (column: ODS-A, solvent gradient: A:B = 0:100 to 100:0 for 45 min, A: CH_3CN with 0.1% TFA, B: H_2O with 0.1% TFA) to obtain **EAT(DHFR)** (3.2 mg, 5.1 μmol, 16%) as a yellow solid.

^{1}H NMR (600 MHz, DMSO-d_6, rt): δ 8.66 (s, 1H), 8.42 (t, J = 6.0 Hz, 1H), 8.26 (d, J = 7.2 Hz, 1H), 7.76–7.73 (m, 4H), 7.39 (d, J = 8.4 Hz, 2H), 7.27 (s, 2H), 6.82 (d, J = 9.0 Hz, 2H), 4.85 (s, 2H), 4.35–4.30 (m, 3H), 3.24 (s, 3H), 2.31–2.28 (m, 2H), 2.10–1.93 (m, 2H).

^{13}C NMR (150 MHz, DMSO-d_6, rt): δ 173.7, 171.7, 166.1, 162.6, 150.7, 148.8, 143.6, 142.5, 128.9, 127.3, 125.6, 121.9, 121.3, 111.1, 54.8, 52.1, 41.6, 39.1, 31.8, 26.5.

Three ^{13}C signals of the pteridine ring could not be observed.

HR-FTMS (ESI): Calcd. For $[M+Na]^+$: m/z = 645.1963; found: 645.1964

EAT(DNP-IgG)

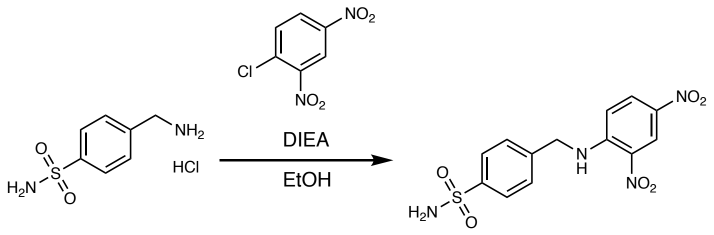

EAT(DNP IgG)

To a solution of homosulfamine hydrochloride (15.6 mg, 70.0 μmol, 1.0 eq) and 1-chloro-2,4-dinitrobenzene (21.2 mg, 105 μmol, 1.5 eq) in dry EtOH (500 μL) was added DIEA (33.2 μL, 210 μmol, 3.0 eq). The reaction mixture was refluxed for 7 h under N_2 atmosphere. The solvent was removed under reduced pressure. The crude product was purified by RP-HPLC (solvent gradient: CH_3CN with 0.1% TFA/H_2O with 0.1% TFA from 35:65 to 80:20 for 30 min) to obtain **EAT(DNP-IgG)** (12.7 mg, 36.0 μmol, 52%) as a yellow solid.

^{1}H NMR (600 MHz, DMSO-d_6, rt): δ 9.47 (t, J = 6.6 Hz, 1H), 8.88 (d, J = 3.0 Hz, 1H), 8.20 (dd, J = 9.6, 2.4 Hz, 1H), 7.79 (d, J = 9.0 Hz, 2H), 7.56 (d, J = 8.4 Hz, 2H), 7.33 (s, 2H), 6.99 (d, J = 9.6 Hz, 1H), 4.85 (d, J = 6.0 Hz, 2H).

^{13}C NMR: (150 MHz, DMSO-d_6, rt): δ 148.0, 143.0, 141.6, 135.2, 130.4, 130.0, 127.2, 126.0, 123.5, 115.5, 45.6.

HR-FTMS (ESI): Calcd. For $[M+Na]^+$: m/z = 375.0370; found: 375.0373

TMR-Gua•TFA

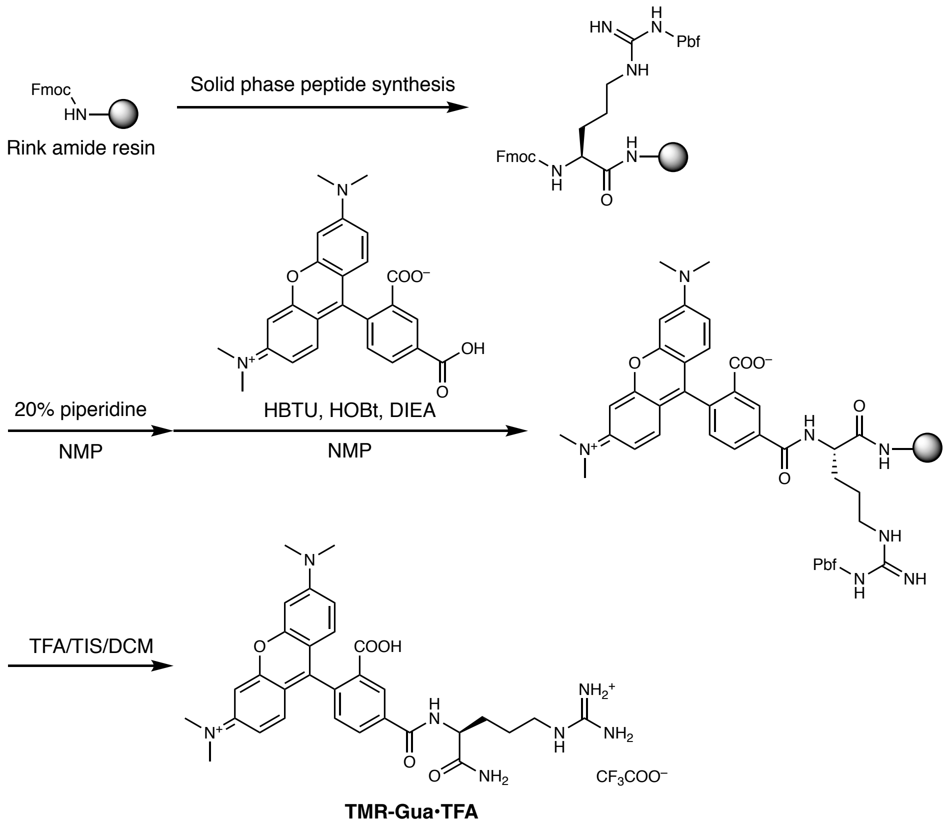

TMR-Gua•TFA was synthesized by solid-phase peptide synthesis using a commercially available rink amide resin (51.0 μmol). The condensation reaction was carried out in the presence of Fmoc-protected amino acids (3.0 eq), 2-(1*H*-benzotriazol-1-yl)-1,1,3,3-tetramethyluronium hexafluorophosphate (HBTU: 3.0 eq), 1-hydroxybenzotriazole hydrate (HOBt·H_2O: 3.0 eq) and diisopropylethylamine (DIEA: 6.0 eq) in 1-methyl-2-pyrrolidinone (NMP). Removal of Fmoc protecting group was performed using NMP solution containing 20% piperidine. After removal of the Fmoc group on the terminal amino group, the resulting free amino group was allowed to react with 5-carboxyl tetramethylrhodamine (3.0 eq) in the presence of HBTU (3.0 eq), HOBt (3.0 eq), and DIEA (6.0 eq) in NMP. Finally, cleavage of the compound from the resin was performed using a cocktail (95:2.5:2.5 TFA/TIS/DCM) at rt for 1.5 h to give crude product. The crude product was purified by RP-HPLC (column: ODS-A, solvent gradient: A:B = 5:95 to 45:55 for 40 min, retention time: 32 min, A: CH_3CN with 0.1% TFA, B: H_2O with 0.1% TFA) to obtain **TMR-Gua•TFA** (22.0 mg, 31.0 μmol, 61%) as a red powder.

^{1}H NMR (400 MHz, CD_3OD, rt): δ 8.83 (d, J = 2.4 Hz, 1H), 8.32 (dd, J = 8.0, 2.4 Hz, 1H), 7.55 (d, J = 8.0 Hz, 1H), 7.14 (d, J = 9.6 Hz, 2H), 7.06 (dd, J = 9.6, 2.4 Hz, 2H), 7.00 (d, J = 2.4 Hz, 2H), 4.73–4.64 (m, 1H), 2.13–1.83 (m, 2H), 1.82–1.62 (m, 2H). Signals of methyl protons of the rhodamine skeleton were overlapped with those of the residual non-deuterated solvent.
HR-FTMS (ESI): Calcd. For $[M+Na]^+$: m/z = 608.2592; found: 608.2590

Fluorescein-modified agarose

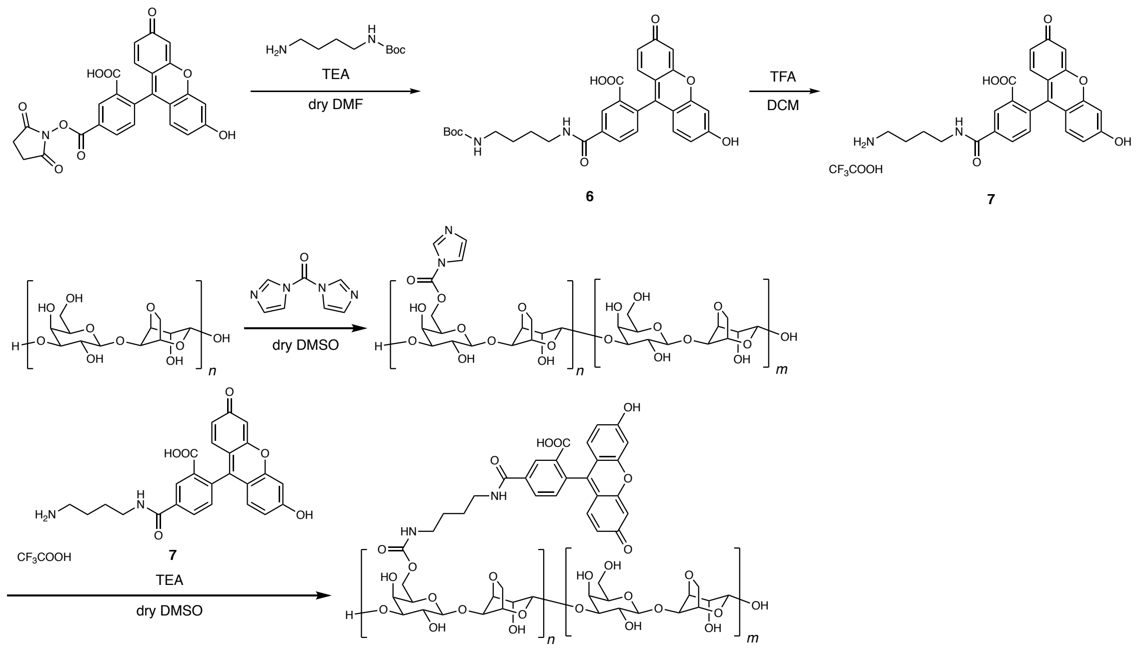

Compound 6

To a dry DMF solution (200 μL) of 5-carboxyfluorescein NHS ester (10.5 mg, 22.2 μmol, 1.0 eq) and *N*-(tert-butoxycarbonyl)-1,4-diaminobutane HCl salt (6.80 mg, 30.3 μmol, 1.36 eq) was added TEA (10 μL, 72 μmol, 3.24 eq). The reaction mixture was allowed to stir at rt for 3 h under N_2 atmosphere. After removal of solvent under reduced pressure, the crude product was purified by silica gel column chromatography ($CHCl_3$:MeOH = 20:1 containing 1 vol% AcOH) to obtain **6** (9.5 mg, 17.4 μmol, 78%) as an orange solid.

^{1}H-NMR (400 MHz, CD_3OD, rt): δ 8.42 (s, 1H), 8.19 (dd, J = 8.0, 1.4 Hz, 1H), 7.30 (d, J = 8.1 Hz, 1H), 6.69 (d, J = 2.3 Hz, 2H), 6.60 (d, J = 8.7 Hz, 2H), 6.54 (dd, J = 8.7, 2.3 Hz, 2H), 3.45 (t, J = 6.8 Hz, 2H), 3.10 (t, J = 6.3 Hz, 2H), 1.70–1.64 (m, 2H), 1.61–1.56 (m, 2H).

HR-FTMS (ESI): Calcd. For $[M+Na]^+$: m/z = 569.1894; found: 569.1893

Compound 7

Compound **6** (9.5 mg, 17.4 μmol) was dissolved in CH_2Cl_2 (1 mL) and TFA (500 μL). The mixture was allowed to stir at rt for 30 min. The crude product was obtained by solvent removal under reduced pressure. The crude product was used for the next reaction without further purification.

Fluorescein modified agarose (FL-agarose)

To a dry DMSO solution (1.5 mL) of agarose (99.6 mg, 310 μmol as monomer unit) was added a dry DMSO solution (15.5 μL) of CDI (0.25 mg, 1.55 μmol, 0.005 eq). The reaction mixture was allowed to stir at rt for 1 h. To this mixture, a DMSO solution (23.3 μL) of **7** (2.33 μmol, 0.0075 eq) and TEA (4.66 μmol, 0.015 eq) was added. The resultant mixture was stirred at rt for 24 h. After dilution by adding H_2O (20 mL), the mixture was dialyzed in H_2O for 6 times (MWCO 1000). The mixture was lyophilized to obtain FL-agarose (81.4 mg) as a pale yellow powder. The modification yield was determined to be 0.015% by UV-vis absorption spectroscopy as shown in supplementary Fig. 40.

Spectral data of the degradation products

F(CF_3)F:

^{1}H NMR (400 MHz, DMSO-d_6, rt): δ 8.82 (d, J = 7.6 Hz, 1H), 7.69 (d, J = 8.0 Hz, 2H), 7.51 (d, J = 8.0 Hz, 2H), 7.33–7.22 (m, 4H), 4.56–4.51 (m, 1H), 4.03–4.00 (m, 1H), 3.22–2.94 (m, 4H).

HR-FTMS (ESI): Calcd. for $[M+Na]^+$: m/z = 381.1421; found: 381.1428

QM-F(CF_3)F:

^{1}H NMR (400 MHz, DMSO-d_6, rt): δ 7.64 (d, J = 8.0 Hz, 2H), 7.37 (d, J = 8.0 Hz, 2H), 7.23 (d, J = 8.0 Hz, 2H), 7.12–7.09 (m, 1H), 7.00 (d, J = 8.0 Hz, 2H), 6.75 (d, J = 8.0 Hz, 2H), 4.70–4.64 (m, 1H), 3.90 (m, 1H), 3.30–3.06 (m, 6H).

HR-FTMS (ESI): Calcd. for $[M+Na]^+$: m/z = 487.1848; found: 487.1839

QM_2-F(CF_3)F:

^{1}H NMR (400 MHz, DMSO-d_6, rt): δ 7.93 (d, J = 7.6 Hz, 1H), 7.69 (d, J = 8.0 Hz, 2H), 7.51 (d, J = 8.0 Hz, 2H), 7.33-7.22 (m, 4H), 4.56–4.51 (m, 1H), 4.03–4.00 (m, 1H), 3.22–2.94 (m, 4H).

HR-FTMS (ESI): Calcd. for $[M+Na]^+$: m/z = 593.2258; found: 593.2264

Supplementary Figures

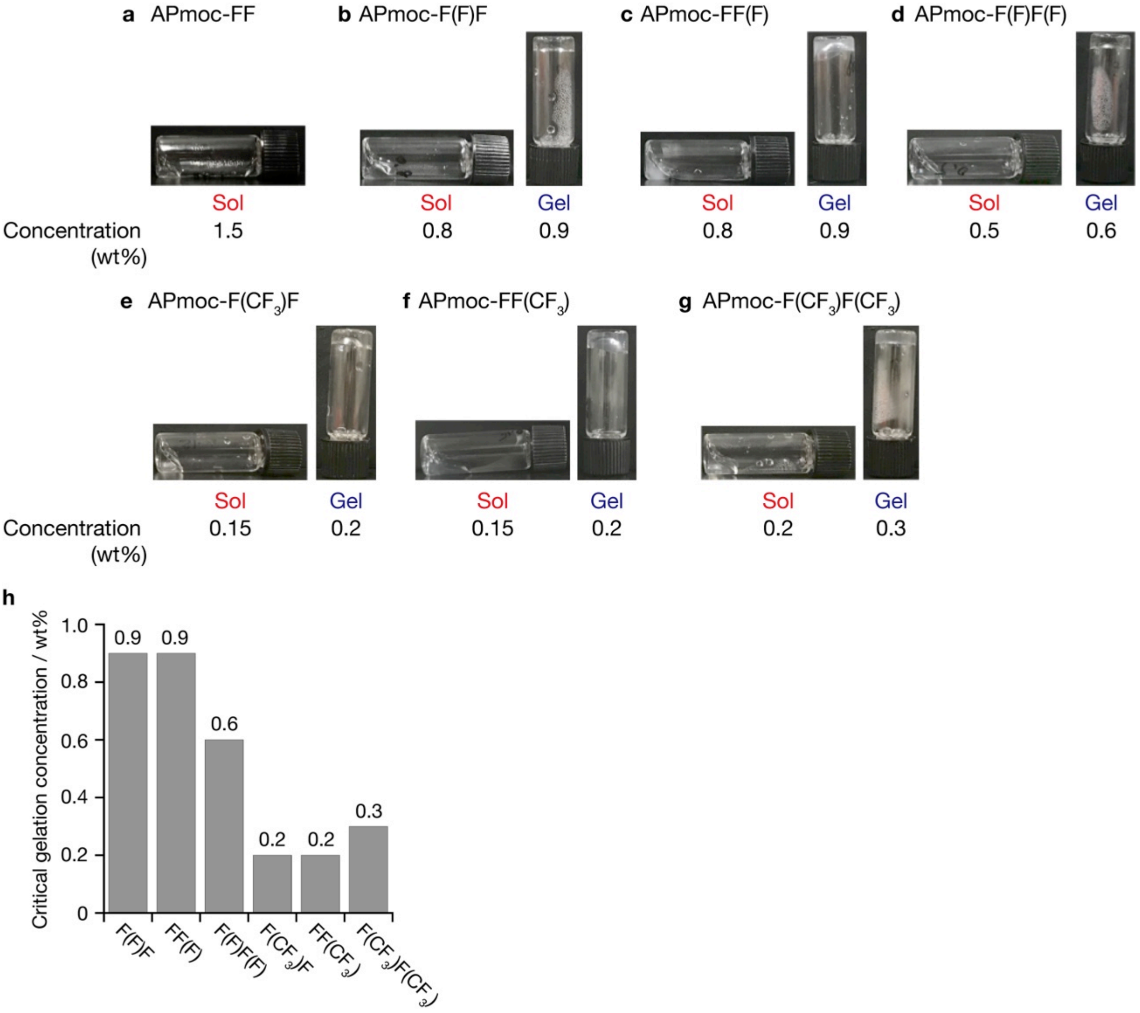

Supplementary Fig. 1. Macroscopic observation of (**a**) **APmoc-FF**, (**b**) **APmoc-F(F)F**, (**c**) **APmoc-FF(F)**, (**d**) **APmoc-F(F)F(F)**, (**e**) **APmoc-F(CF_3)F**, (**f**) **APmoc-FF(CF_3)**, and (**g**) **APmoc-F(CF_3)F(CF_3)**. (**h**) Critical gelation concentrations of APmoc gelators. Condition: 100 mM HEPES, pH 8.0.

a

b

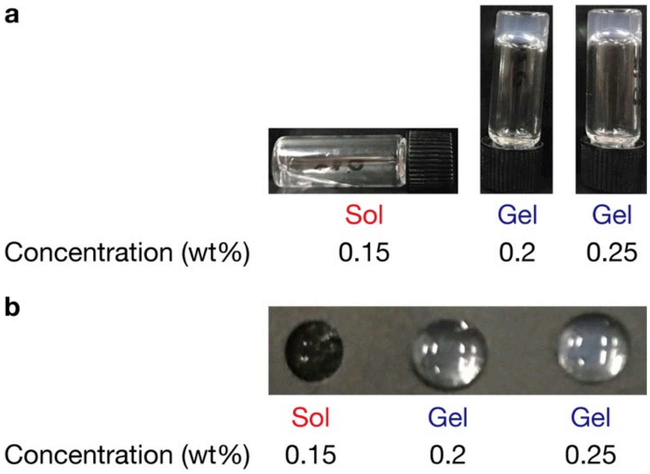

Supplementary Fig. 2. Determination of critical gelation concentration of **APmoc-F(CF_3)F** by (**a**) a tube inversion test and (**b**) a paper absorption test. Condition: 100 mM HEPES, pH 8.0.

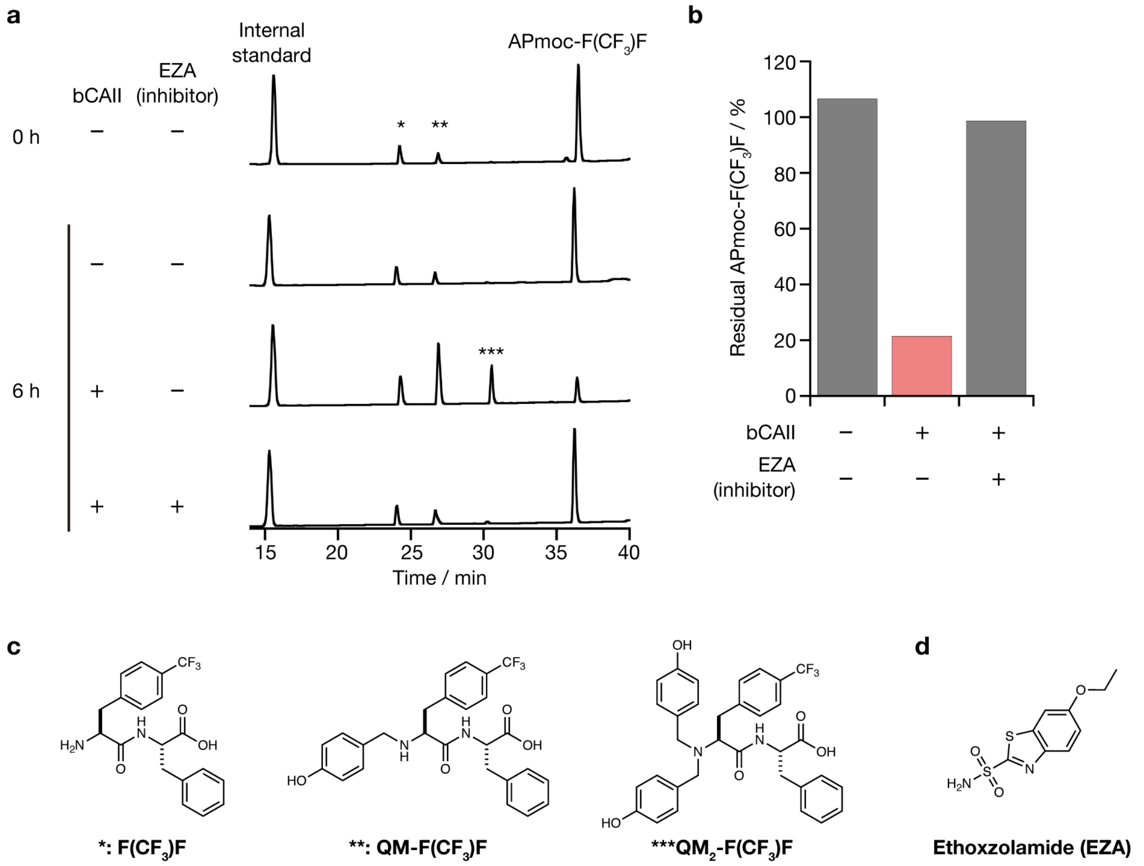

Supplementary Fig. 3. (**a**) HPLC charts of **APmoc-F(CF$_3$)F** before and after addition of bCAII with/without a competitive inhibitor, EZA. Internal standard: terephthalic acid. (**b**) Residual ratios of **APmoc-F(CF$_3$)F** determined by HPLC charts. The signal intensity before bCAII addition was used as 100% standard. Conditions: [**APmoc-F(CF$_3$)F**] = 0.35 wt%, [bCAII] = 10 μM, [EZA] = 100 μM, 100 mM HEPES, pH 8.0, 25 ºC, 0 or 6 h. V_{gel}:$V_{stimulus}$ = 10:1 (**c**) Plausible chemical structures of the degradation products determined by ESI-MS and ^{1}H NMR. (**d**) Chemical structure of ethoxzolamide (EZA).

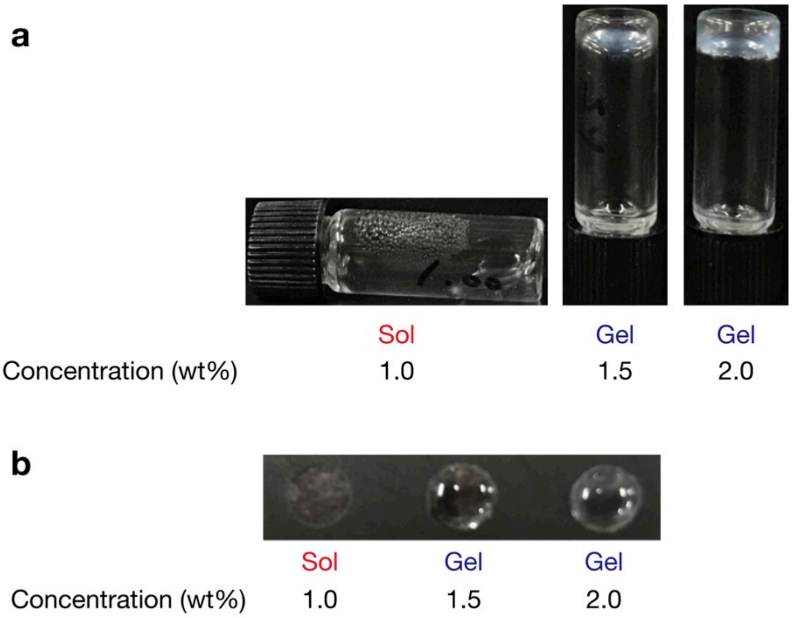

Supplementary Fig. 4. Determination of critical gelation concentration of **Bz-FF** by (**a**) a tube inversion test and (**b**) a paper absorption test. Condition: 100 mM HEPES, pH 8.0.

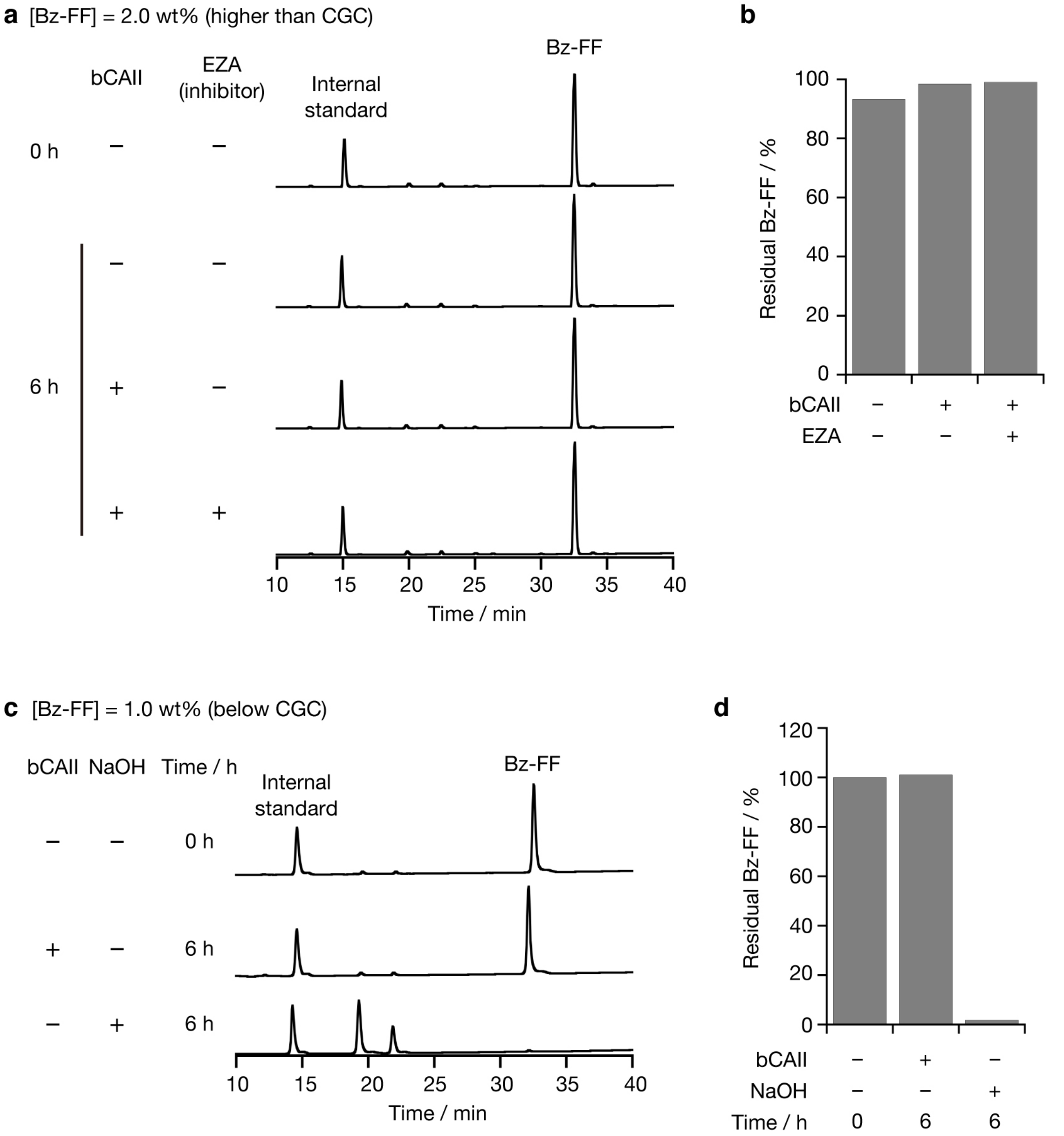

Supplementary Fig. 5. (**a**, **c**) HPLC charts of **Bz-FF** ((**a**) above CGC (2.0 wt%) and (**c**) below CGC (1.0 wt%)) before and after addition of bCAII with/without a competitive inhibitor, EZA. Internal standard: terephthalic acid. (**b, d**) Residual ratios of **Bz-FF** determined by HPLC charts of supplementary Fig. (**b**) 5a and (**d**) 5c. The signal intensity before bCAII addition was used as 100% standard. Conditions: [**Bz-FF**] = 2.0 or 1.0 wt% (42 or 21 mM), [bCAII] = 10 μM, [EZA] = 0 or 100 μM, [NaOH] = 0 or 100 mM, 100 mM HEPES, pH 8.0, 25 ºC, 6 h.

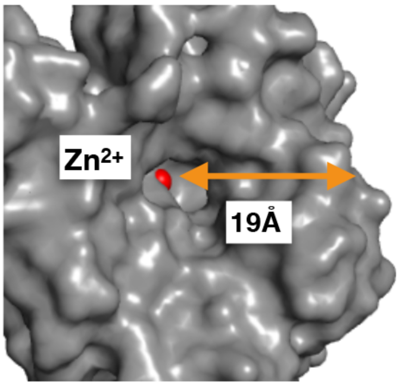

Supplementary Fig. 6. Crystal structure of bCAII. An orange arrow shows a distance from the catalytic Zn^{2+} center to the surface. PDB ID: 1V9I.

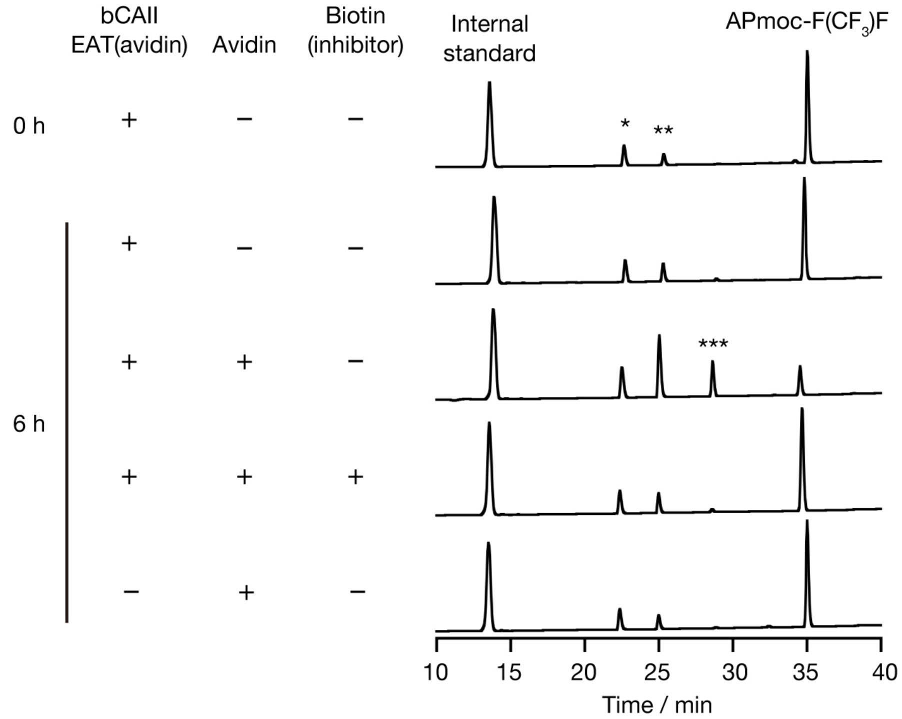

Supplementary Fig. 7. HPLC charts of **APmoc-F(CF_3)F** hydrogel containing bCAII/**EAT(avidin)** before and after addition of avidin with/without a competitive inhibitor, biotin. The signal intensity before avidin addition was used as 100% standard. Internal standard: terephthalic acid. The background hydrolysis of the acetyl group occurred probably because the enzymatic activity of bCAII was not perfectly inhibited under the this condition for the optimal protein response. When the amount of EAT is increased, the minimal amount of protein analytes required for a gel-sol transition would be increased, which indicates the lowering of the protein sensitivity. The background hydrolysis can be ignored because the response mechanism relies on a threshold-type gel-sol transition. Condition: [**APmoc-F(CF_3)F**] = 0.32 wt% (5.6 mM), [bCAII] = 10 μM, [**EAT(avidin)**] = 20 μM, [avidin] = 20 μM, [biotin] = 0 or 120 μM, 100 mM HEPES, pH 8.0, 25 ºC, 6 h, V_{gel}:$V_{stimulus}$ = 10:1. *: **F(CF_3)F**, **: **QM-F(CF_3)F**, ***: **QM_2-F(CF_3)F**.

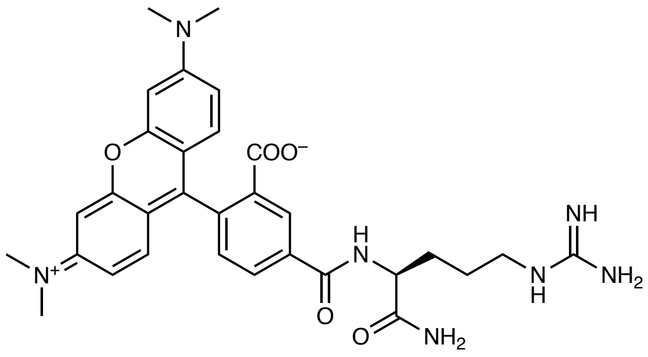

TMR-Gua

Supplementary Fig. 8. Chemical structure of the fluorescent probe, **TMR-Gua**

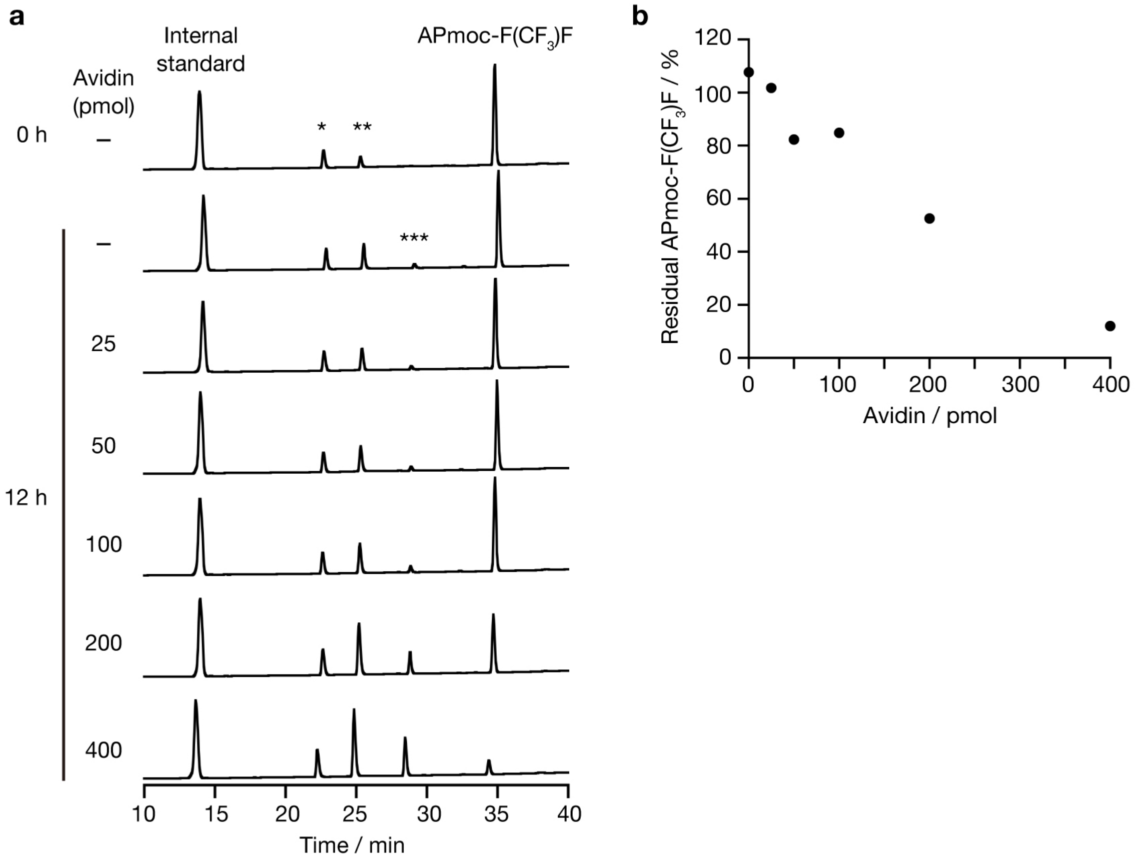

Supplementary Fig. 9. (**a**) HPLC charts of **APmoc-F(CF$_3$)F** containing bCAII/**EAT(avidin)** before and after addition of various amounts of avidin. Internal standard: terephthalic acid. (**b**) Plot of the remained amount of **APmoc-F(CF$_3$)F** against avidin concentration. The signal intensity before avidin addition was used as 100% standard. Condition: [**APmoc-F(CF$_3$)F**] = 0.32 wt% (5.6 mM), [bCAII] = 10 μM, [**EAT(avidin)**] = 20 μM, [avidin] = 0, 1.25, 2.5, 5.0, 10, and 20 μM, 100 mM HEPES, pH 8.0, 25 ºC, 12 h, V_{gel}:$V_{stimulus}$ = 10:1. *: **F(CF$_3$)F**, **: **QM- F(CF$_3$)F**, ***: **QM$_2$-F(CF$_3$)F**.

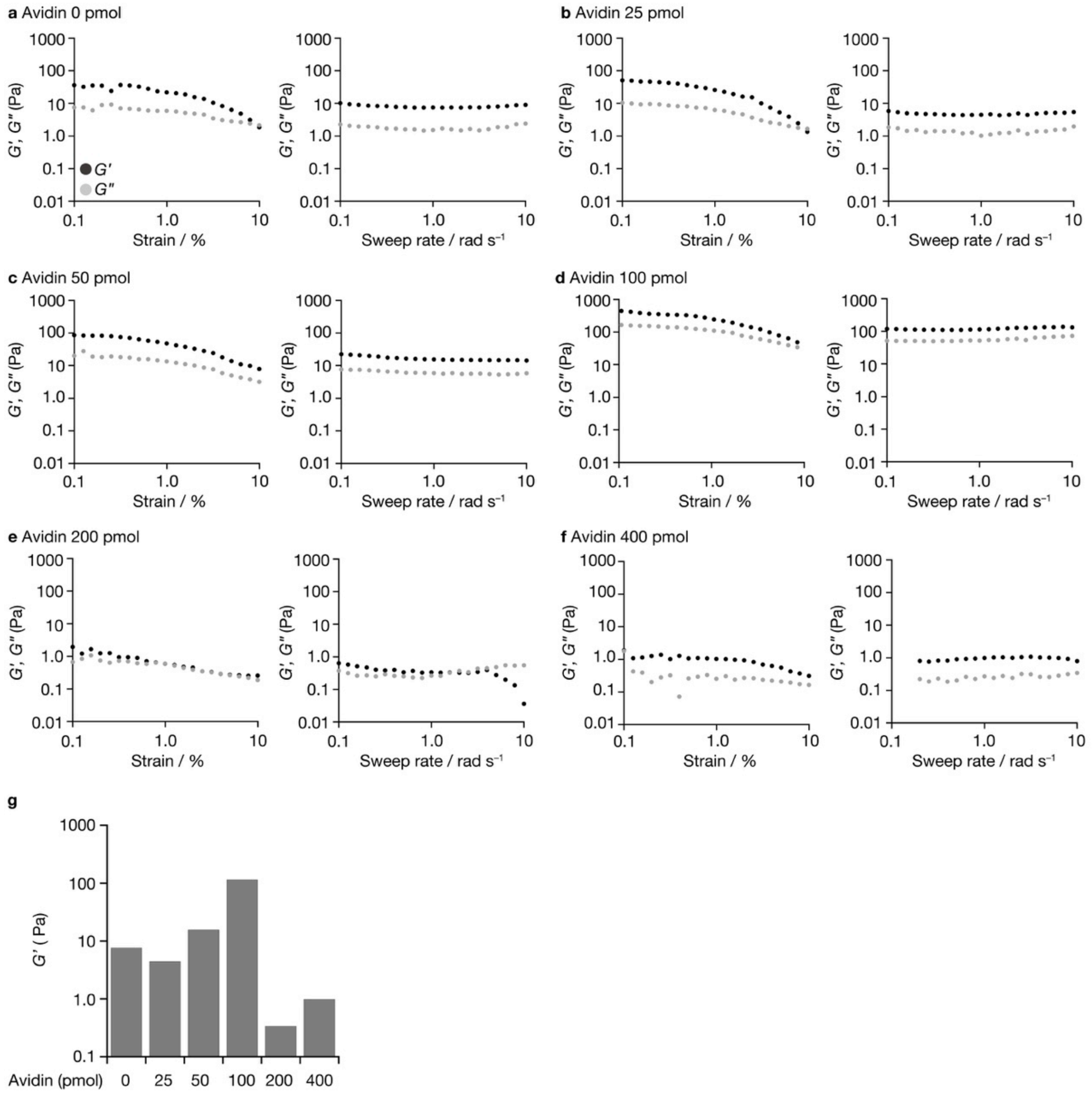

Supplementary Fig. 10. (Left) Strain sweep and (right) frequency sweep rheological analyses of **APmoc-F(CF$_3$)F** containing bCAII/**EAT(avidin)** before and after addition of various amounts of avidin (**a**: 0, **b**: 25, **c**: 50, **d**: 100, **e**: 200, **f**: 400 pmol). Frequency for strain sweep: 10 rad/s. Strain amplitude for frequency sweep: 1%. *G′*: storage shear modulus, *G″*: loss shear modulus. (**g**) Storage modulus (*G′*) values of the hydrogel after addition of avidin. Condition: [**APmoc-F(CF$_3$)F**] = 0.32 wt% (5.6 mM), [bCAII] = 10 μM, [**EAT(avidin)**] = 20 μM, [avidin] = 0, 1.25, 2.5, 5.0, 10, and 20 μM, 100 mM HEPES, pH 8.0, 25 ºC, 12 h, V_{gel}:$V_{stimulus}$ = 10:1.

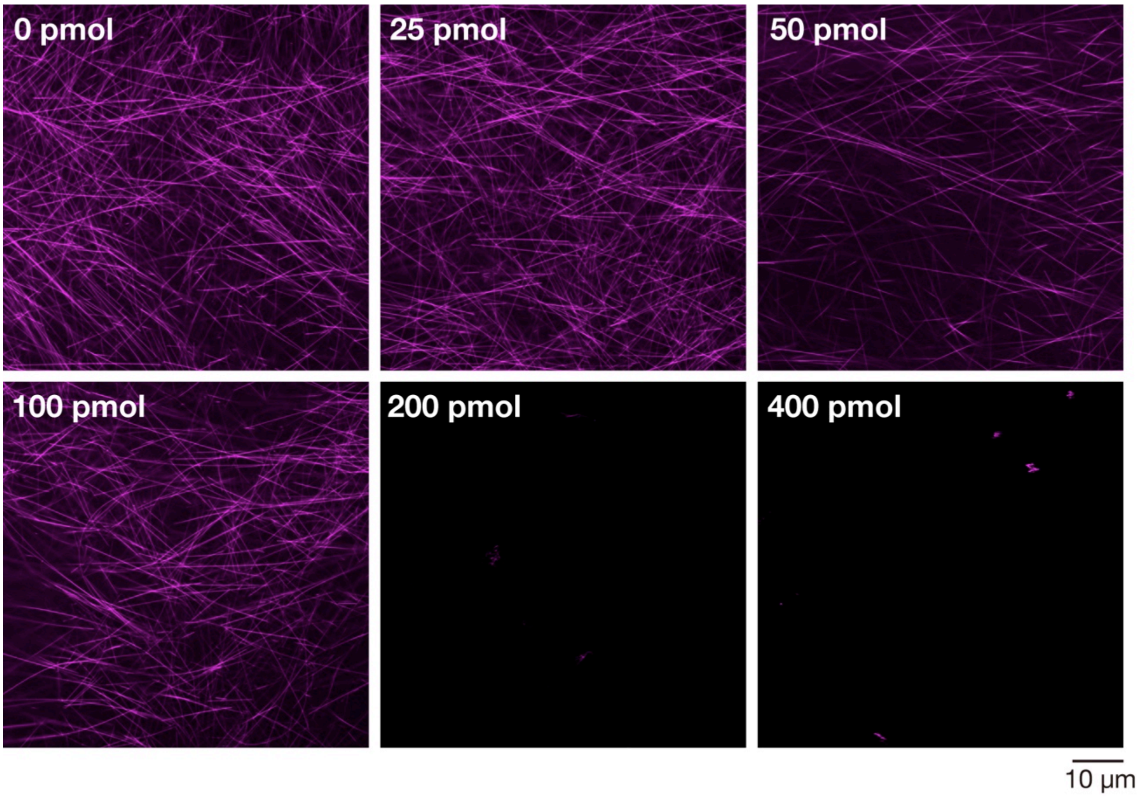

Supplementary Fig. 11. CLSM imaging of **APmoc-F(CF$_3$)F** containing bCAII/**EAT(avidin)**/**TMR-Gua** before and after addition of various amounts of avidin. Condition: [**APmoc-F(CF$_3$)F**] = 0.32 wt% (5.6 mM), [**TMR-Gua**] = 10 µM, [bCAII] = 10 µM, [**EAT(avidin)**] = 20 µM, [avidin] = 0, 1.25, 2.5, 5.0, 10, and 20 µM, 100 mM HEPES, pH 8.0, 25 ºC, 12 h, V_{gel}:$V_{stimulus}$ = 10:1.

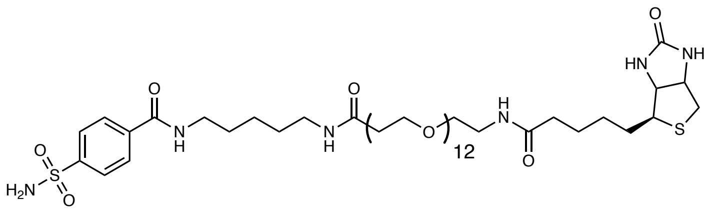

Supplementary Fig. 12. Chemical structure of **EAT(avidin, long).**

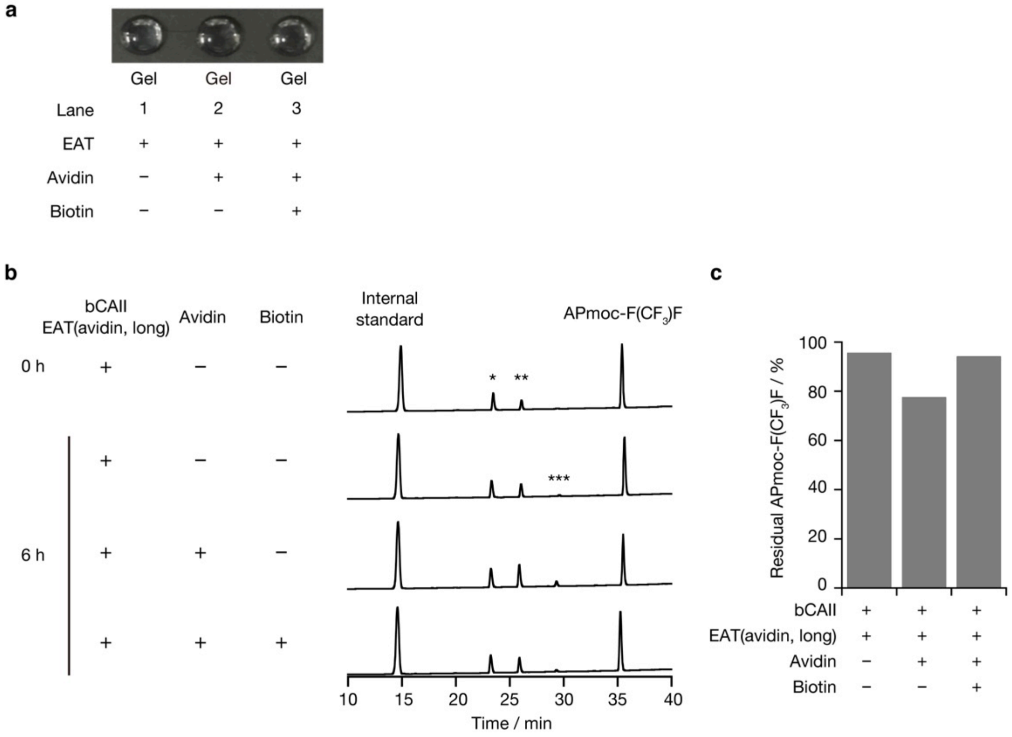

Supplementary Fig. 13. (**a**) Avidin response on the hydrogel array chip with **EAT(avidin, long)**. (**b**) HPLC charts of **APmoc-F(CF_3)F** hydrogel with bCAII/**EAT(avidin, long)** before and after addition of avidin with/without biotin. Internal standard: terephthalic acid. (**c**) Residual ratios of **APmoc-F(CF_3)F** determined by HPLC. The signal intensity before avidin addition was used as 100% standard. Condition: [**APmoc-F(CF_3)F**] = 0.32 wt% (5.6 mM), [bCAII] = 10 μM, [**EAT(avidin, long)**] = 20 μM, [avidin] = 20 μM, [biotin] = 0 or 120 μM in 100 mM HEPES, pH 8.0, 6 h, 25 ºC. V_{gel}:$V_{stimulus}$ = 10:1. *: **F(CF_3)F**, **: **QM-F(CF_3)F**, ***: **QM_2-F(CF_3)F**.

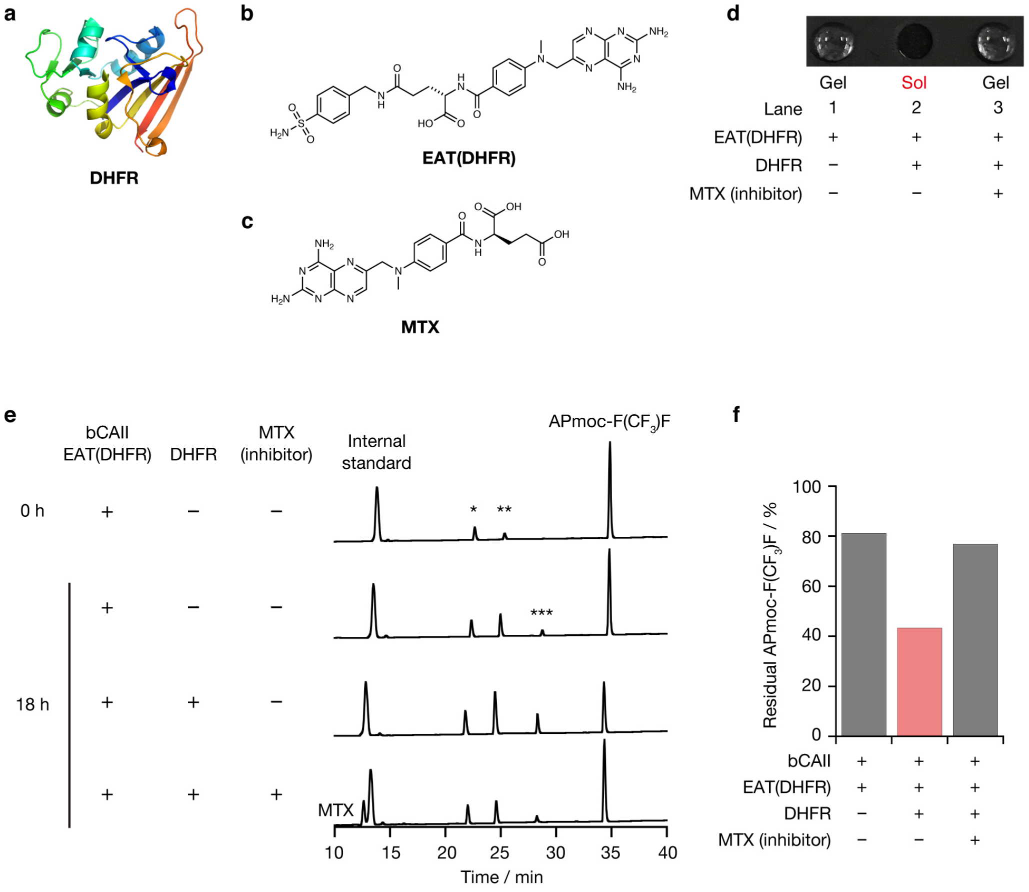

Supplementary Fig. 14. (**a**) Crystal structure of DHFR. PDB ID: 3DAU. Chemical structures of (**b**) **EAT(DHFR)** and (**c**) MTX, a competitive inhibitor. (**d**) DHFR response on the hydrogel array chip. (**e**) HPLC charts of **APmoc-F(CF$_3$)F** hydrogel with bCAII/**EAT(DHFR)** before and after addition of DHFR with/without a DHFR ligand, MTX. Internal standard: terephthalic acid. The signal intensity before DHFR addition was used as 100% standard. (**f**) Residual ratios of **APmoc-F(CF$_3$)F** determined by HPLC. Conditions: [**APmoc-F(CF$_3$)F**] = 0.32 wt% (5.6 mM), [bCAII] = 10 μM, [**EAT(DHFR)**] = 45 μM, [DHFR] = 45 μM, [MTX] = 0 or 450 μM, 100 mM HEPES, pH 8.0, 25 ºC, 0 or 18 h. V_{gel}:$V_{stimulus}$ = 10:1. *: **F(CF$_3$)F**, **: **QM-F(CF$_3$)F**, ***: **QM$_2$-F(CF$_3$)F**.

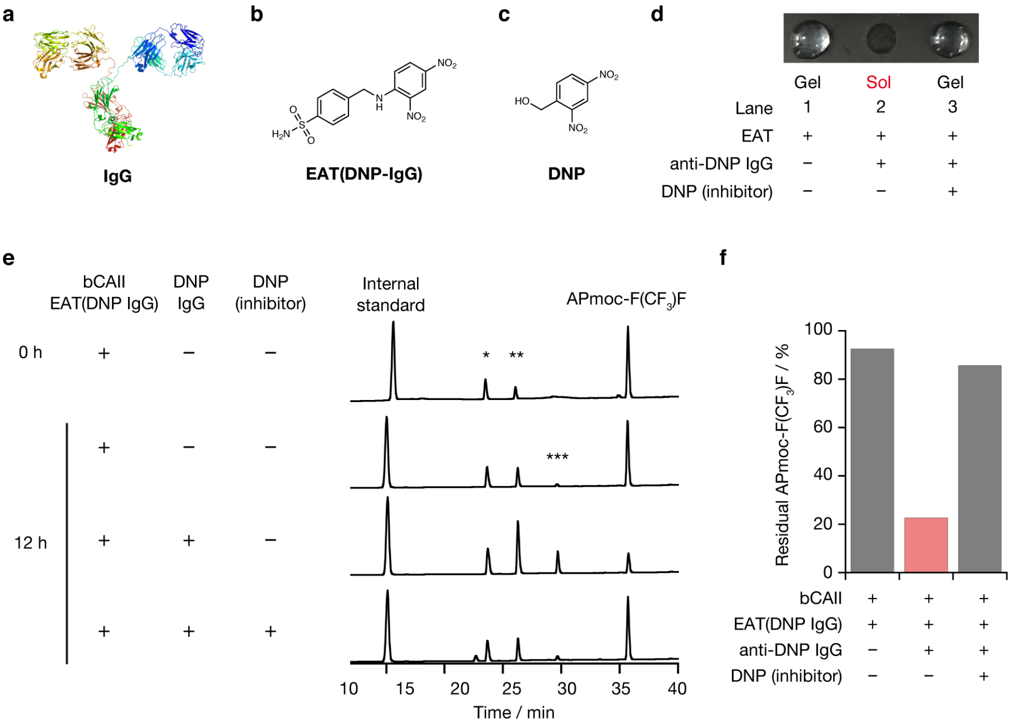

Supplementary Fig. 15. (**a**) Crystal structure of IgG. PDB ID: 1IGT. Chemical structures of (**b**) **EAT(DNP-IgG)** and (**c**) DNP, a competitive inhibitor. (**d**) Anti-DNP IgG response on the hydrogel array chip. (**e**) HPLC charts of **APmoc-F(CF$_3$)F** hydrogel with bCAII/**EAT(DNP-IgG)** before and after addition of anti-DNP IgG with/without DNP. Internal standard: terephthalic acid. (**f**) Residual ratios of **APmoc-F(CF$_3$)F** determined by HPLC. The signal intensity before anti-DNP IgG addition was used as 100% standard. Conditions: [**APmoc-F(CF$_3$)F**] = 0.32 wt%, [bCAII] = 10 μM, [**EAT(DNP-IgG)**] = 15 μM, [anti-DNP IgG] = 15 μM, [DNP] = 0 or 150 μM, 100 mM HEPES, pH 8.0, rt, 0 or 12 h. V_{gel}:$V_{stimulus}$ = 10:1. *: **F(CF$_3$)F**, **: **QM-F(CF$_3$)F**, ***: **QM$_2$-F(CF$_3$)F**.

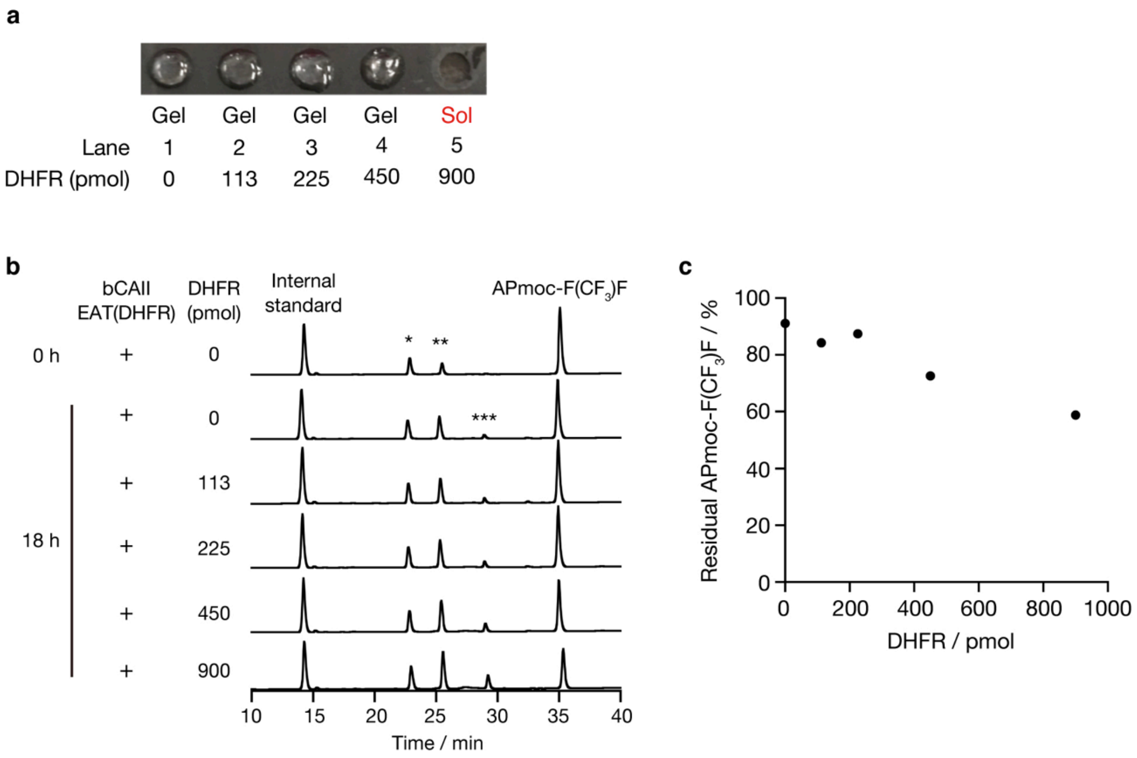

Supplementary Fig. 16. (**a**) Determination of the detection threshold by the hydrogel array chip. (**b**) HPLC charts of **APmoc-F(CF$_3$)F** containing bCAII/**EAT(DHFR)** before and after addition of various amounts of DHFR. (**c**) Plot of the remained amount of **APmoc-F(CF$_3$)F** against DHFR concentration. Internal standard: terephthalic acid. The signal intensity before DHFR addition was used as 100% standard. Conditions: [**APmoc-F(CF$_3$)F**] = 0.32 wt% (5.6 mM), [bCAII] = 10 µM, [**EAT(DHFR)**] = 45 µM, [DHFR] = 0, 5.65, 11.3, 22.5, 45 µM, 100 mM HEPES, pH 8.0, 25 ºC, 0 or 18 h. V_{gel}:$V_{stimulus}$ = 10:1. *: **F(CF$_3$)F**, **: **QM-F(CF$_3$)F**, ***: **QM$_2$-F(CF$_3$)F**.

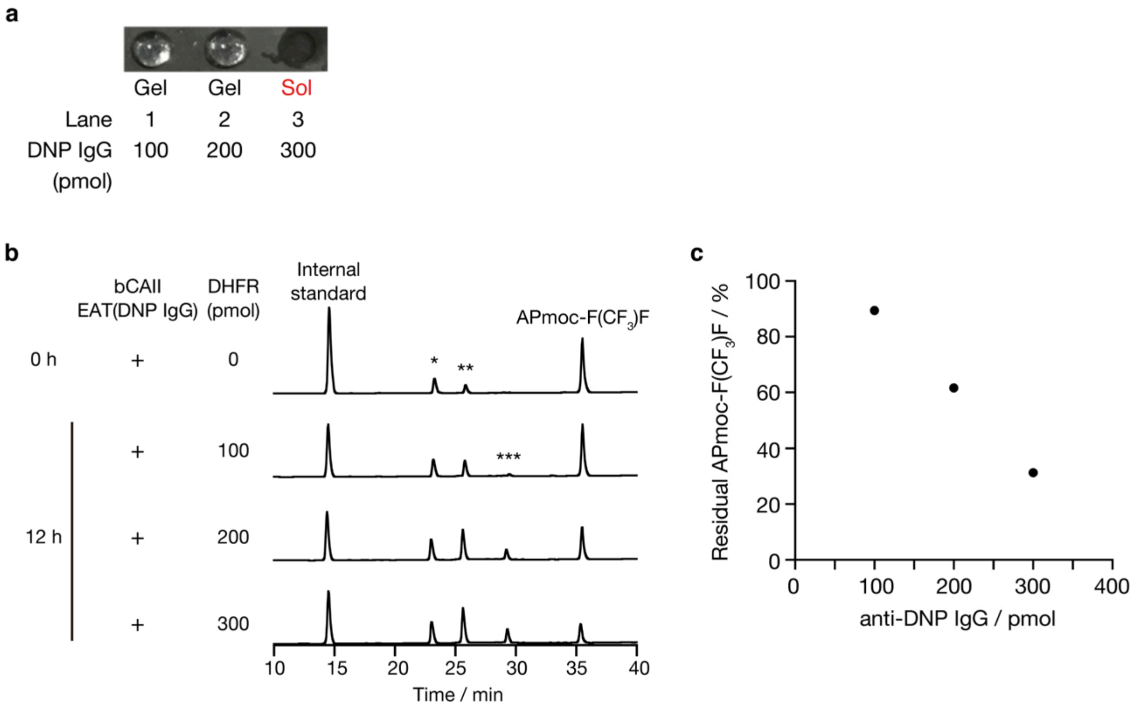

Supplementary Fig. 17. (**a**) Determination of the detection threshold by the hydrogel array chip. (**b**) HPLC charts of **APmoc-F(CF$_3$)F** containing bCAII/**EAT(DNP-IgG)** before and after addition of various amounts of anti-DNP IgG. Internal standard: terephthalic acid. (**c**) Plot of the remained amount of **APmoc-F(CF$_3$)F** against anti-DNP IgG concentration. The signal intensity before anti-DNP IgG addition was used as 100% standard. Conditions: [**APmoc-F(CF$_3$)F**] = 0.32 wt%, [bCAII] = 10 μM, [**EAT(DNP-IgG)**] = 15 μM, [anti-DNP IgG] = 0, 5, 10, 15 μM, 100 mM HEPES, pH 8.0, rt, 0 or 12 h. V_{gel}:$V_{stimulus}$ = 10:1. *: **F(CF$_3$)F**, **: **QM-F(CF$_3$)F**, ***: **QM$_2$-F(CF$_3$)F**.

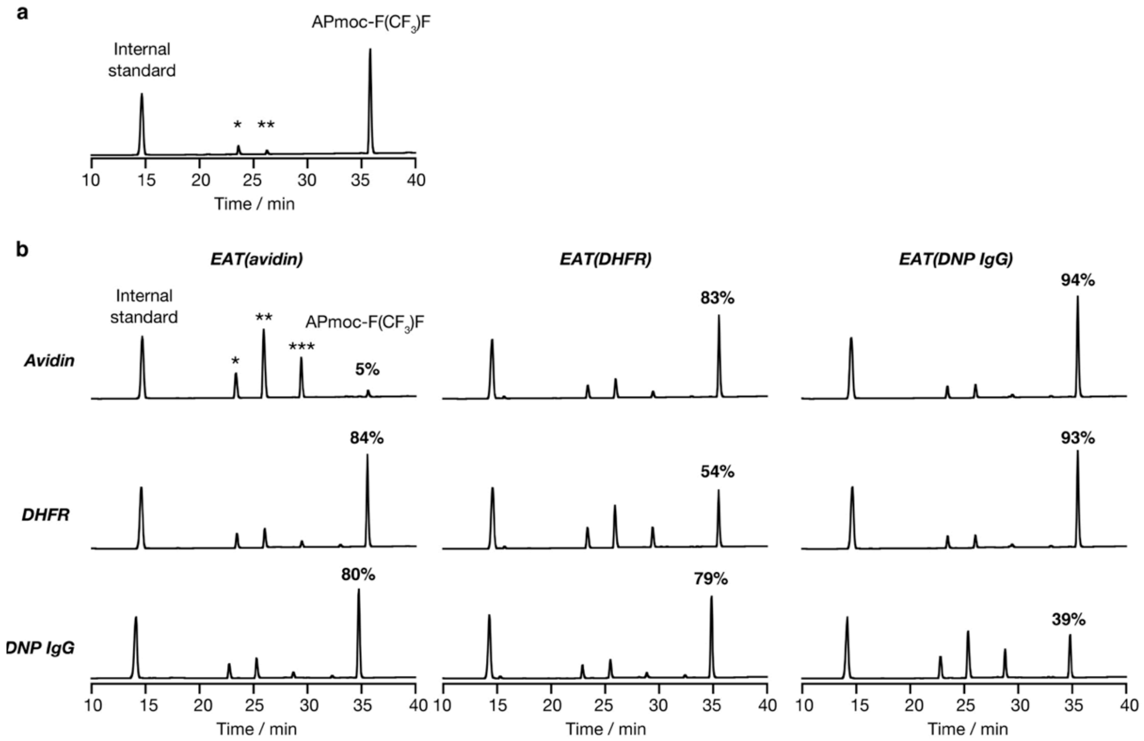

Supplementary Fig. 18. HPLC charts of **APmoc-F(CF$_3$)F** containing bCAII/EAT (**a**) before and (**b**) after addition of protein analytes. Internal standard: terephthalic acid. The signal intensity before addition of protein analytes was used as 100% standard. Conditions: [**APmoc-F(CF$_3$)F**] = 0.32 wt% (5.6 mM), [bCAII] = 10 μM, [**EAT(avidin)**] = 20 μM, [**EAT(DHFR)**] = 45 μM, [**EAT(DNP-IgG)**] = 15 μM, [avidin] = 20 μM, [DHFR] = 45 μM, [anti-DNP IgG] = 15 μM, 100 mM HEPES, pH 8.0, rt, 0 or 18 h. V_{gel}:$V_{stimulus}$ = 10:1. *: **F(CF$_3$)F**, **: **QM-F(CF$_3$)F**, ***: **QM$_2$-F(CF$_3$)F**.

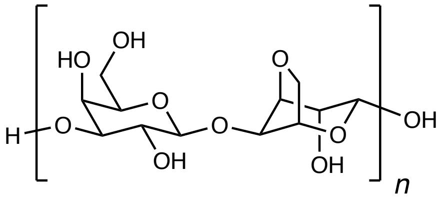

Supplementary Fig. 19. Chemical structure of agarose.

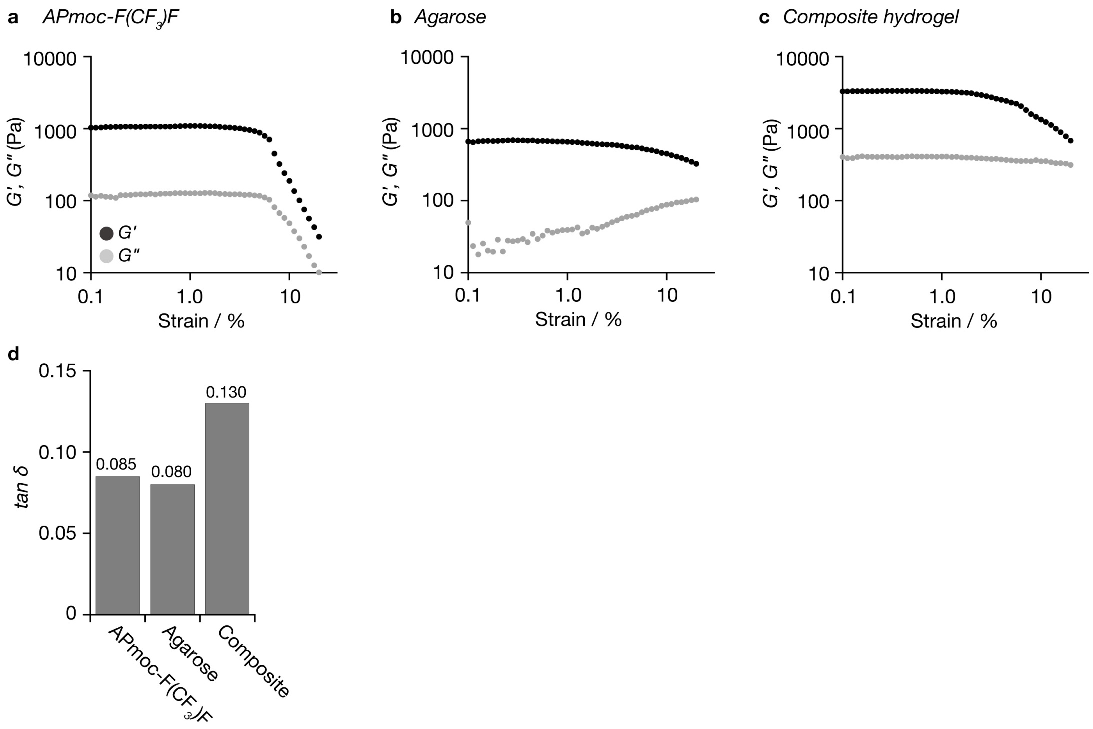

Supplementary Fig. 20. (**a**–**c**) Strain sweep rheological properties of the (**a**) **APmoc-F(CF$_3$)F**, (**b**) agarose, and (**c**) composite hydrogels. Frequency for strain sweep: 10 rad/s. *G'*: storage shear modulus, *G''*: loss shear modulus. (**d**) tan δ values of **APmoc-F(CF$_3$)F**, agarose, and composite gels. Frequency: 0.2 rad/s, strain amplitude: 1%. Condition: [**APmoc-F(CF$_3$)F**] = 0.6 wt%, [agarose] = 0.5 wt%, 100 mM HEPES, pH 8.0.

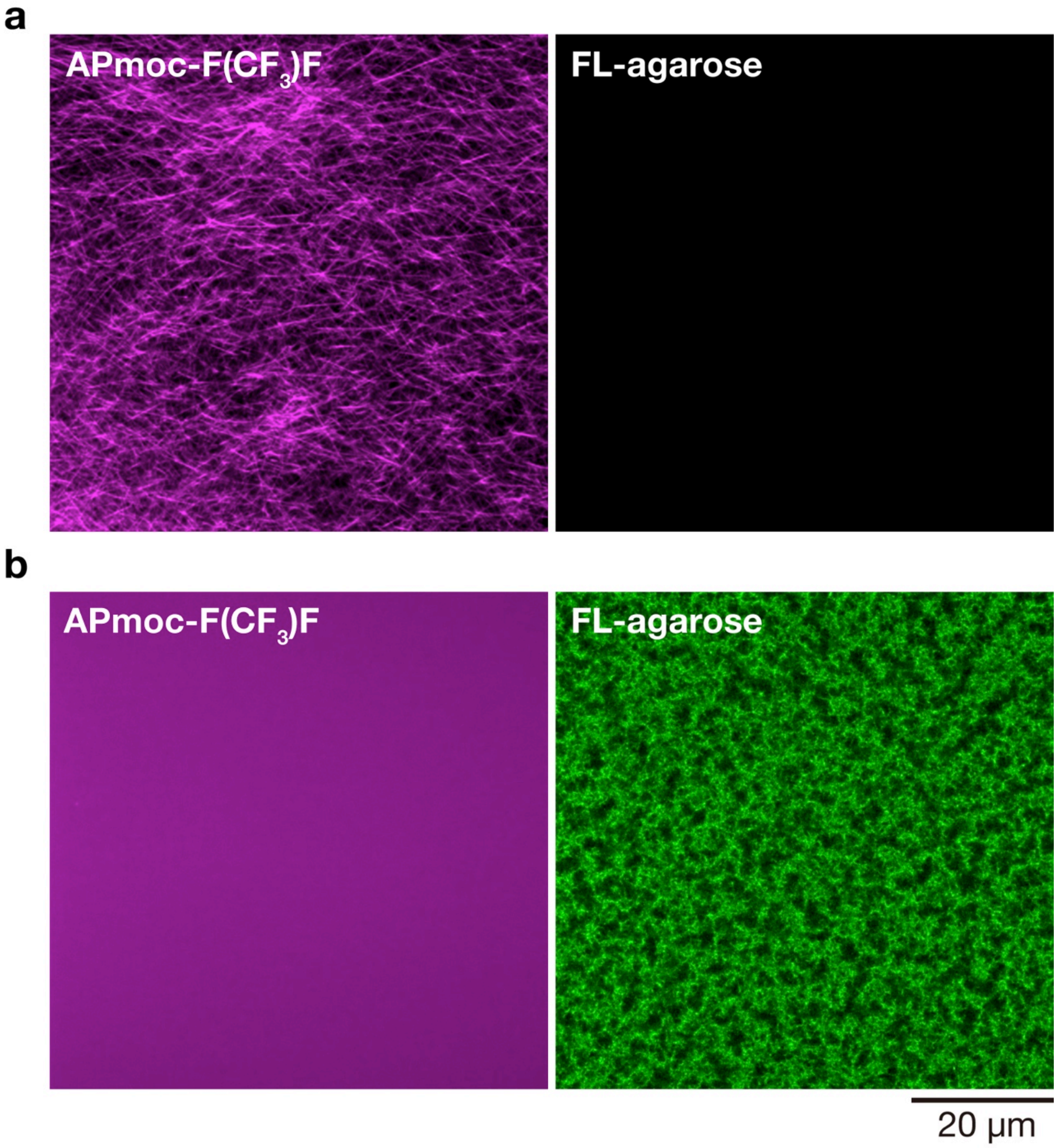

Supplementary Fig. 21. High-resolution Airyscan CLSM images of (**a**) **APmoc-F(CF$_3$)F** hydrogel and (**b**) FL-agarose hydrogel in the presence of **TMR-gua**. Left: TMR channel, right: fluorescein channel. Condition: [**APmoc-F(CF$_3$)F**] = 0.6 wt%, [FL-agarose] = 0.5 wt%, [**TMR-Gua**] = 10 μM, 100 mM HEPES, pH 8.0.

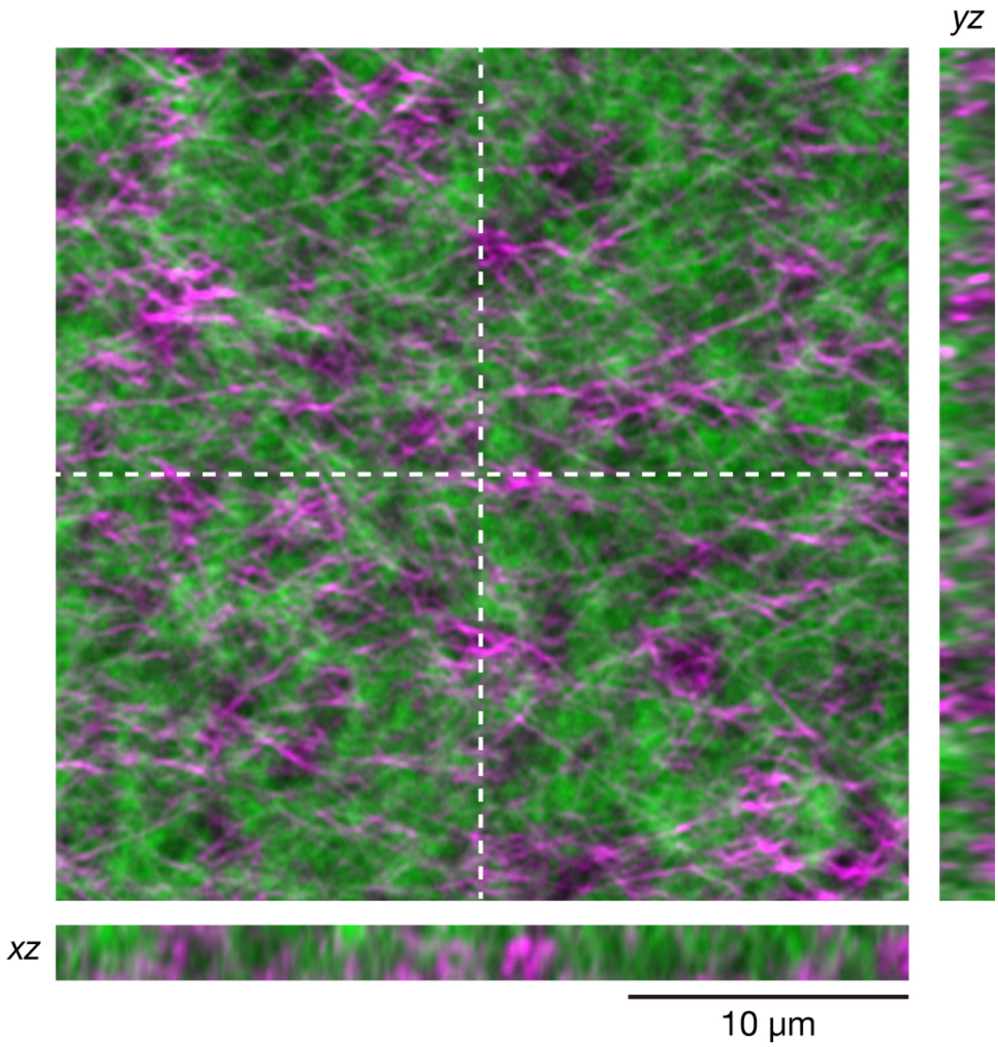

Supplementary Fig. 22. Orthosteric projections of the composite hydrogel. **APmoc-F(CF$_3$)F** nanofibers and FL-agarose did not overlap along the *z* axis direction. Magenta: TMR channel (**APmoc-F(CF$_3$)F** fibers), green: fluorescein channel (FL-agarose). Condition: [**APmoc-F(CF$_3$)F**] = 0.6 wt%, [FL-agarose] = 0.5 wt%, [**TMR-Gua**] = 10 μM, 100 mM HEPES, pH 8.0.

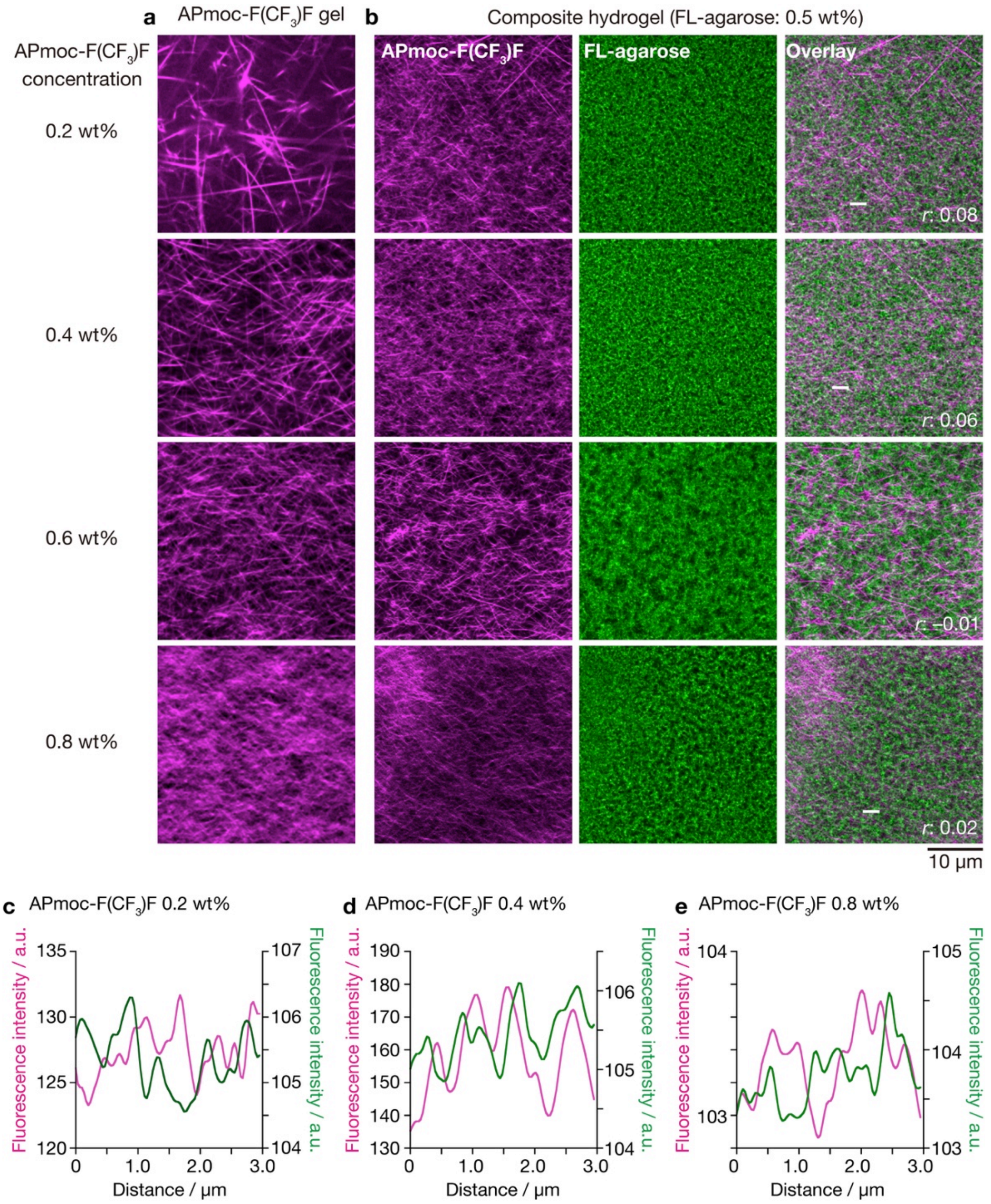

Supplementary Fig. 23. Concentration dependency of Airyscan CLSM imaging of **APmoc-F(CF$_3$)F** in the (**a**) absence and (**b**) presence of FL-agarose. This concentration dependence CLSM analysis confirmed that the orthogonal fiber network structure was formed over a wide range of **APmoc-F(CF$_3$)F** concentrations. Condition: [**APmoc-F(CF$_3$)F**] = 0.2~0.8 wt%, [FL-agarose] = 0.5 wt%, [**TMR-Gua**] = 10 μM, 100 mM HEPES, pH 8.0. *r*: Pearson's correlation coefficient. (**c**–**e**) Line plot analyses along white lines shown in the overlay images. Magenta: **APmoc-F(CF$_3$)F**, green: FL-agarose.

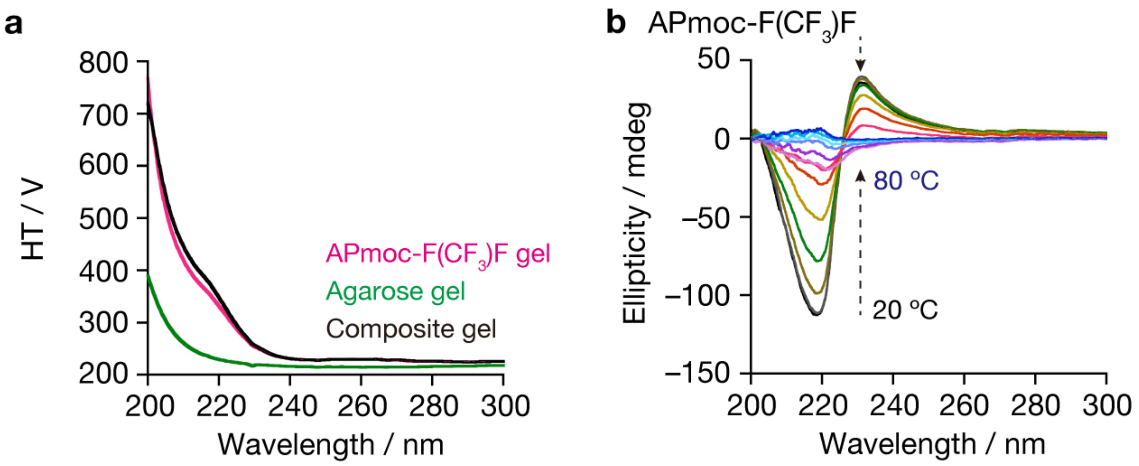

Supplementary Fig. 24. (**a**) HT voltage data of the (magenta) **APmoc-F(CF$_3$)F**, (green) agarose, and (black) composite hydrogels. Temperature: 25 ºC. (**b**) Temperature-dependence CD spectra of the **APmoc-F(CF$_3$)F** hydrogels. Temperature interval: 5 ºC. Condition: [**APmoc-F(CF$_3$)F**] = 0.6 wt%, [agarose] = 0.5 wt%, 100 mM HEPES, pH 8.0, optical length: 0.05 mm.

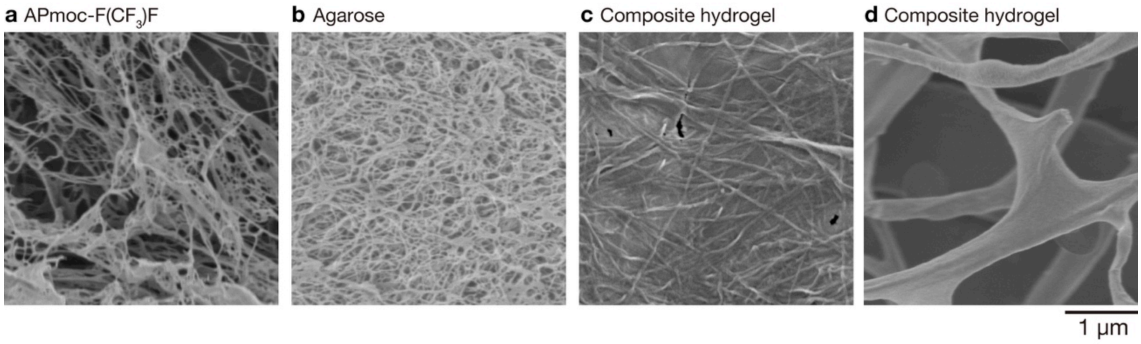

Supplementary Fig. 25. FE-SEM images of (**a**) the **APmoc-F(CF_3)F**, (**b**) agarose, and (**c**, **d**) composite hydrogels. In the composite hydrogel, two different morphologies (two-dimensional sheet structure and thick bundled structure) were observed. As shown in supplementary Fig. 26, the orthogonality of **APmoc-F(CF_3)F** and agarose retained in H_2O as confirmed by CLSM imaging. Condition: [**APmoc-F(CF_3)F**] = 0.6 wt%, [agarose] = 0.5 wt%, H_2O.

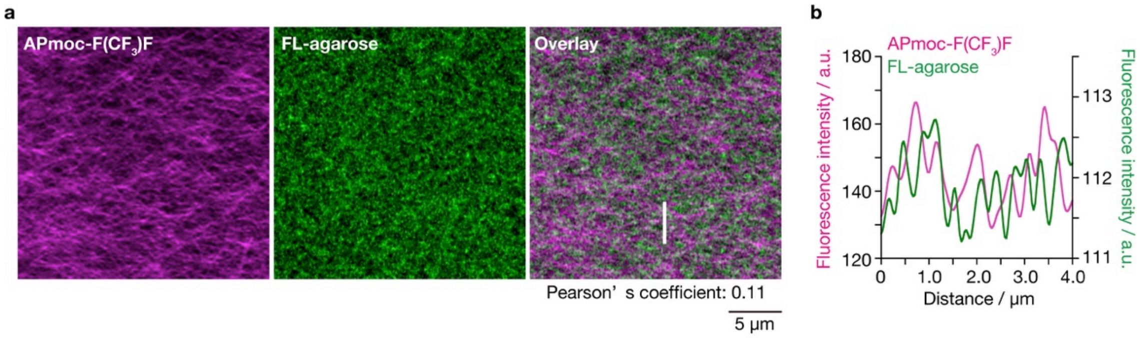

Supplementary Fig. 26. (**a**) CLSM imaging of the composite hydrogel in H_2O. Right: **APmoc-F(CF$_3$)F**, middle: FL-agarose, and right: overlay image. (**b**) Line plot analysis along a white line in the overlay image. The line plot analysis and Pearson's coefficient showed **APmoc-F(CF$_3$)F** and agarose formed the orthogonal network structure. Condition: [**APmoc-F(CF$_3$)F**] = 0.6 wt%, [FL-agarose] = 0.5 wt%, [**TMR-Gua**] = 10 μM, H_2O. *r*: Pearson's correlation coefficient. Magenta: **APmoc-F(CF$_3$)F**, green: FL-agarose.

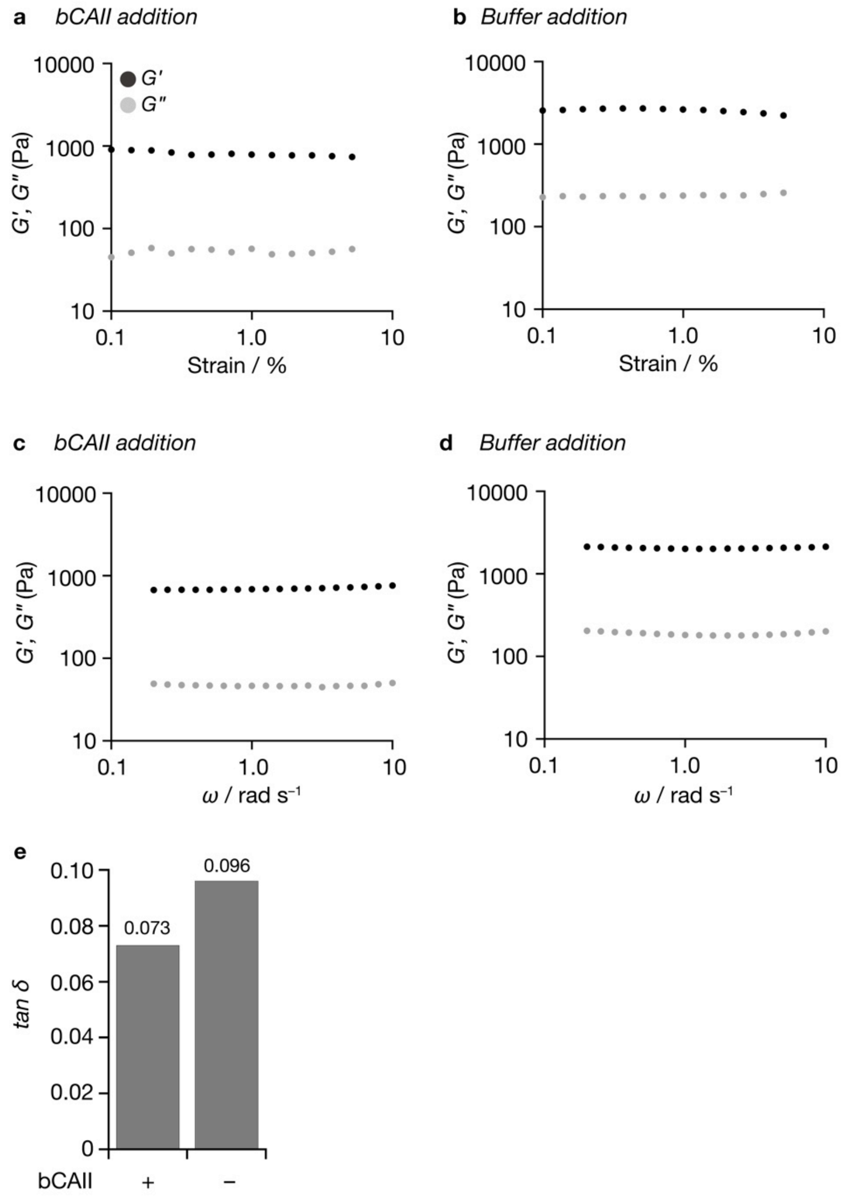

Supplementary Fig. 27. Rheological analysis of bCAII response of the composite hydrogel. (**a**, **b**) Strain sweep and (**c**, **d**) frequency sweep rheological properties of the composite hydrogels after addition of (**a**, **c**) bCAII solution and (**b**, **d**) 100 mM HEPES (pH 8.0). Frequency for strain sweep: 10 rad/s. Strain amplitude for frequency sweep: 1%. *G'*: storage shear modulus, *G"*: loss shear modulus. (**e**) tan δ values of the composite hydrogels after addition of bCAII solution and buffer. Frequency: 0.2 rad/s, strain: 1%. Condition: [**APmoc-F(CF_3)F**] = 0.6 wt%, [agarose] = 0.5 wt%, [bCAII] = 30 μM, 100 mM HEPES, pH 8.0. V_{gel}:$V_{stimulus}$ = 10:1.

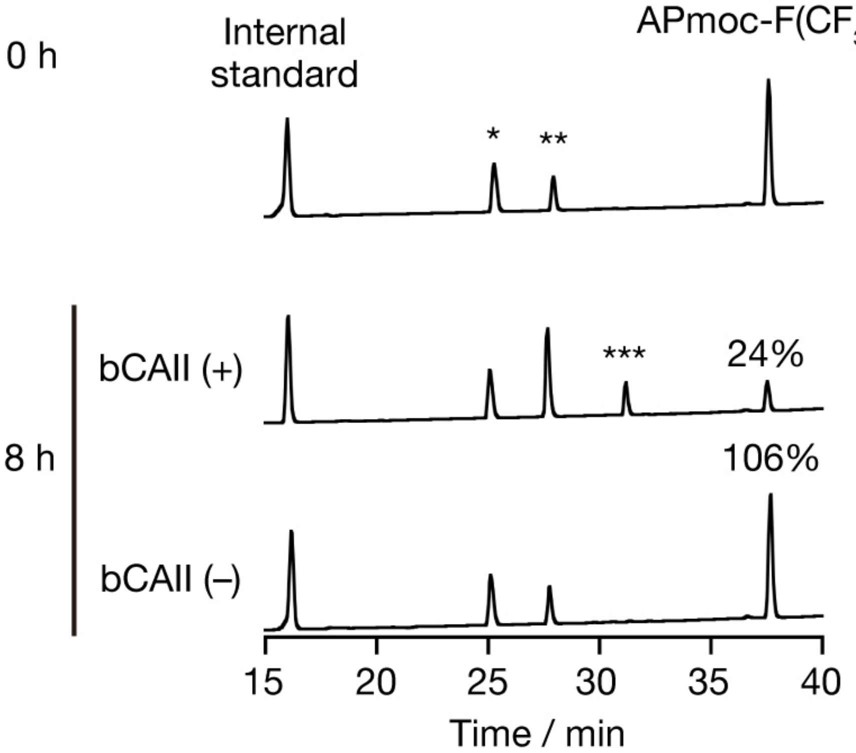

Supplementary Fig. 28. HPLC charts of the composite hydrogel with and without treatment of bCAII. Internal standard: terephthalic acid. The signal intensity before bCAII addition was used as 100% standard. Condition: [**APmoc-F(CF_3)F**] = 0.6 wt%, [agarose] = 0.5 wt%, [bCAII] = 30 μM, 100 mM HEPES, pH 8.0, 25 ºC, 8 h. V_{gel}:$V_{stimulus}$ = 10:1. *: **F(CF_3)F**, **: **QM-F(CF_3)F**, ***: **QM_2-F(CF_3)F**.

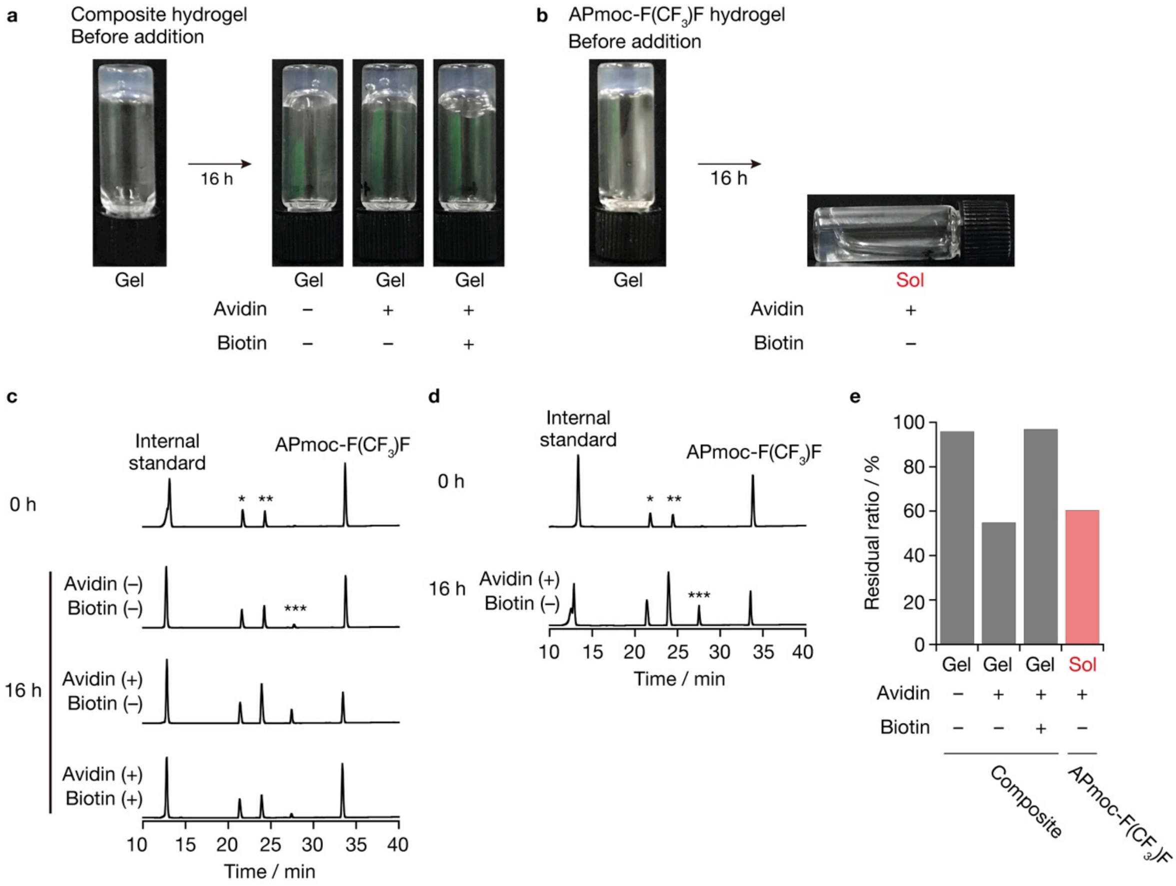

Supplementary Fig. 29. Avidin response of the composite hydrogel. (**a**, **b**) Photographs of (**a**) the composite and (**b**) **APmoc-F(CF$_3$)F** hydrogels containing bCAII/**EAT(avidin)** before and after addition of avidin. (**c, d**) HPLC charts of (**c**) the composite and (**d**) agarose hydrogels containing bCAII/**EAT(avidin)** before and after addition of avidin. Internal standard: terephthalic acid. (**e**) Residual ratio of **APmoc-F(CF$_3$)F** determined by the HPLC charts. The signal intensity before avidin addition was used as 100% standard. Condition: [**APmoc-F(CF$_3$)F**] = 0.6 wt%, [agarose] = 0.5 wt%, [bCAII] = 10 μM, [**EAT(avidin)**] = 20 μM, [avidin] = 20 μM, 100 mM HEPES, pH 8.0, 25 ºC, 16 h. *: **F(CF$_3$)F**, **: **QM-F(CF$_3$)F**, ***: **QM$_2$-F(CF$_3$)F**.

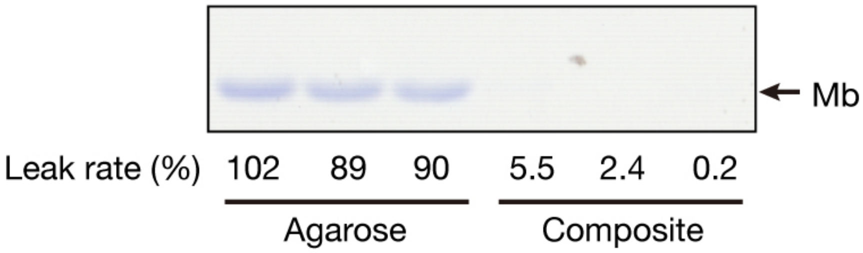

Supplementary Fig. 30. SDS-PAGE analysis of Mb release from the agarose and composite hydrogels. Condition: [**APmoc-F(CF$_3$)F**] = 0.6 wt%, [agarose] = 0.5 wt%, [Mb] = 36 μM, 100 mM HEPES, pH 8.0, 3 h.

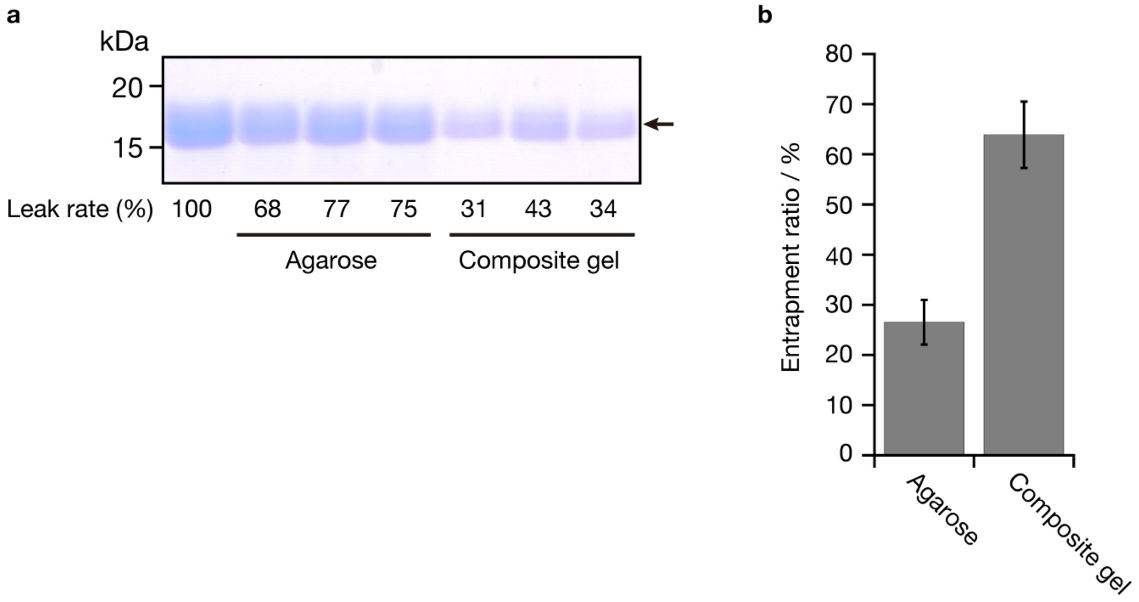

Supplementary Fig. 31. Entrapment of Ax647-Mb in the agarose and composite hydrogels. (**a**) SDS-PAGE analysis of supernatant. The leftmost lane shows the standard for 100% release. (**b**) Averaged entrapment ratio of Ax647-Mb. Condition: [FL-agarose] = 0.5 wt%, [**APmoc-F(CF_3)F**] = 0 or 0.6 wt%, [Ax647-Mb] = 36 μM, 100 mM HEPES, pH 8.0, 25 ºC, 3 h. The data represent the mean ± standard deviation.

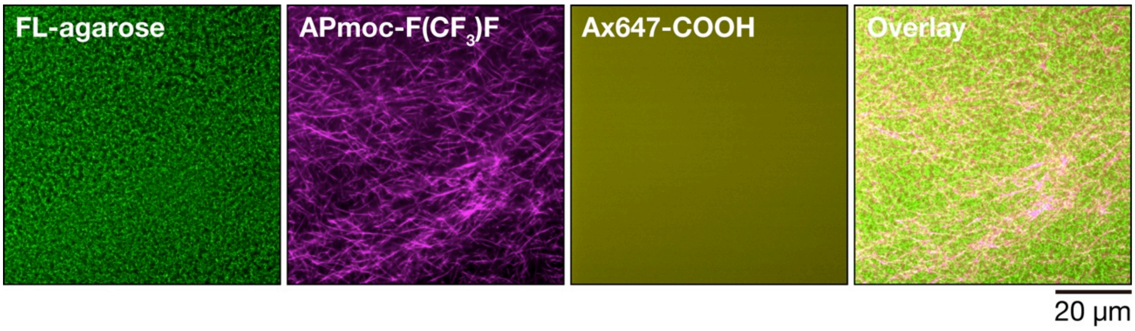

Supplementary Fig. 32. High resolution Airyscan CLSM images of Alexa Fluor 647 carboxylic acid (Ax647-COOH)⊂FL-agarose/**APmoc-F(CF$_3$)F**/**TMR-Gua**. Condition: [FL-agarose] = 0.5 wt%, [**APmoc-F(CF$_3$)F**] = 0.6 wt%, [**TMR-Gua**] = 10 μM, [Alexa Fluor 647 carboxylic acid] = 39 μM, 100 mM HEPES, pH 8.0. Alexa Fluor 647 carboxylic acid was purchased from Thermo Fisher, A33084.

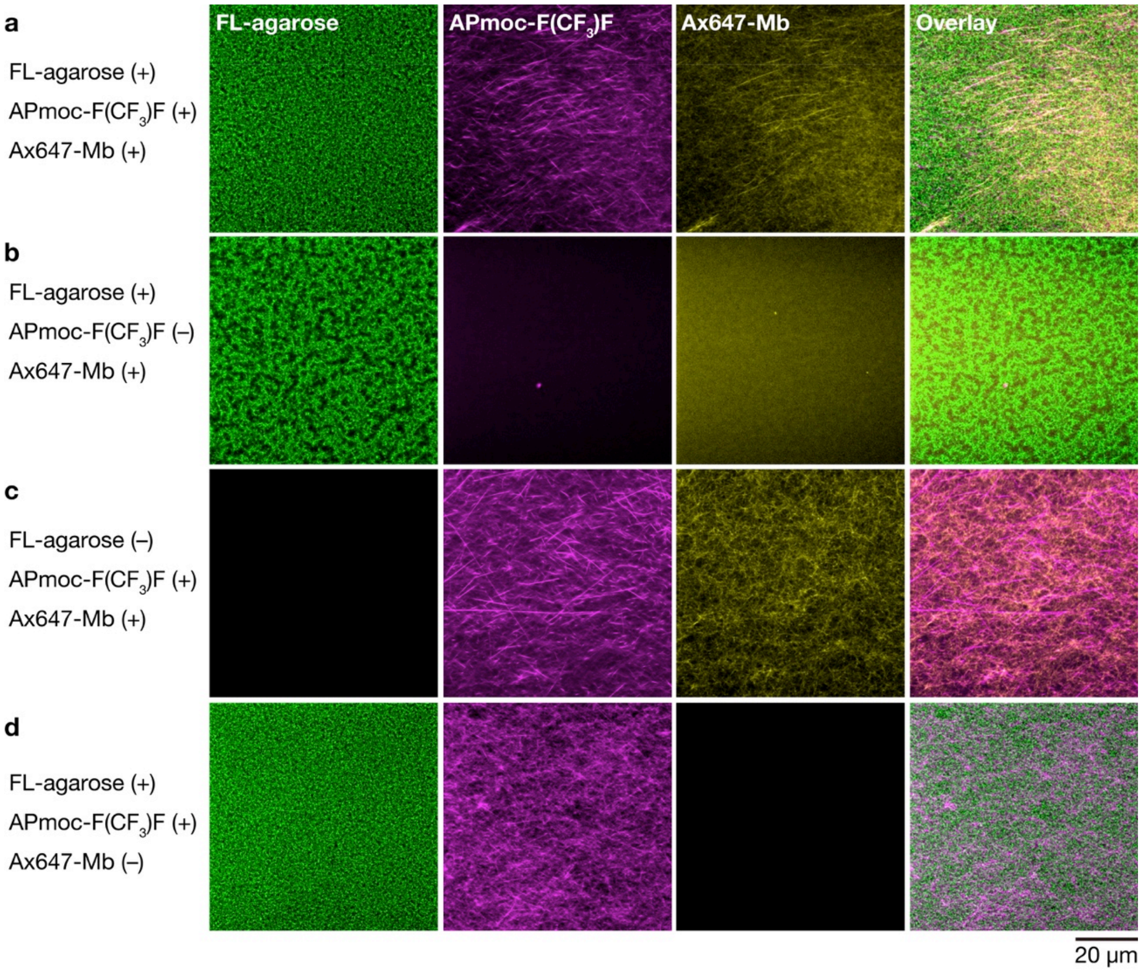

Supplementary Fig. 33. High-resolution Airyscan CLSM images of (**a**) Ax647-Mb⊂FL-agarose/**APmoc-F(CF_3)F**/**TMR-Gua**, (**b**) Ax647-Mb⊂FL-agarose/**TMR-Gua**, (**c**) Ax647-Mb⊂**APmoc-F(CF_3)F**/**TMR-Gua**, (**d**) FL-agarose/**APmoc-F(CF_3)F**/**TMR-Gua**. Green: fluorescein channel, magenta: TMR channel, yellow: Alexa Fluor 647 channel. Condition: [FL-agarose] = 0.5 wt%, [**APmoc-F(CF_3)F**] = 0.6 wt%, [**TMR-Gua**] = 10 μM, [Ax647-Mb] = 36 μM, 100 mM HEPES, pH 8.0.

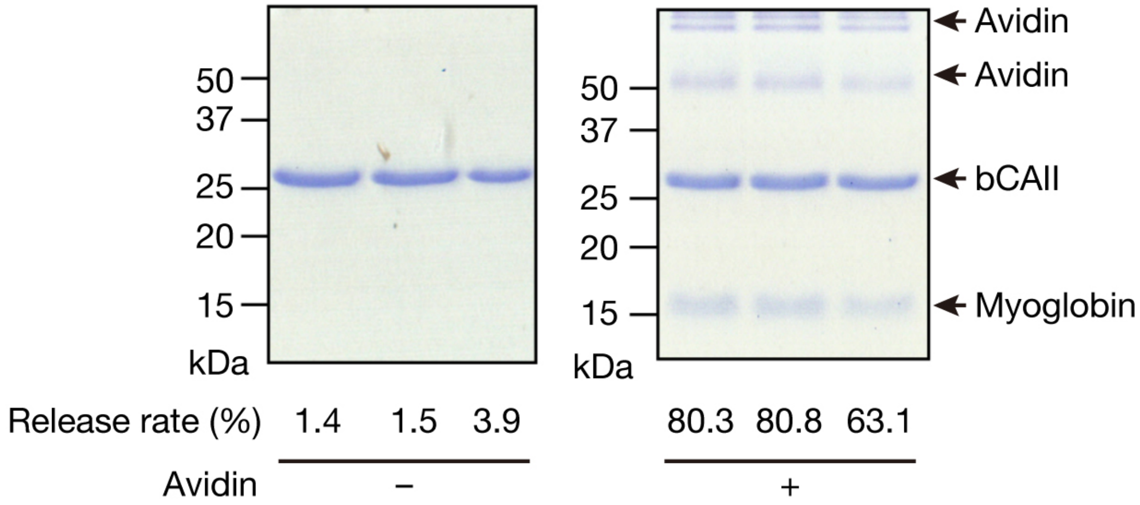

Supplementary Fig. 34. SDS-PAGE analysis of avidin-responsive Mb release from the composite hydrogel containing bCAII/**EAT(avidin)**. Condition: [**APmoc-F(CF$_3$)F**] = 0.6 wt%, [agarose] = 0.5 wt%, [bCAII] = 10 μM, [**EAT(avidin)**] = 20 μM, [Mb] = 36 μM, 100 mM HEPES, pH 8.0, 3 h.

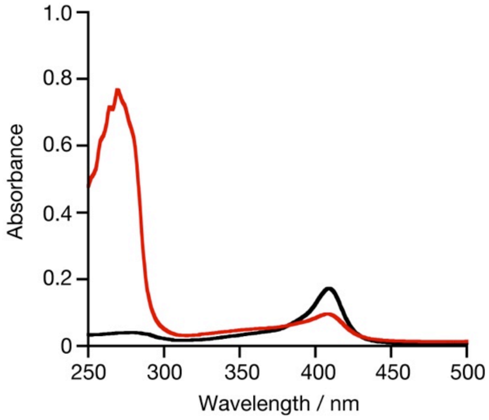

Supplementary Fig. 35. UV-vis absorption spectra of (black) myoglobin (1.8 μM) and (red) a supernatant of the release experiment. The supernatant solution was diluted by 10-fold by HEPES buffer. Condition: 100 mM HEPES, pH 8.0, 25 ºC, optical length: 10 mm.

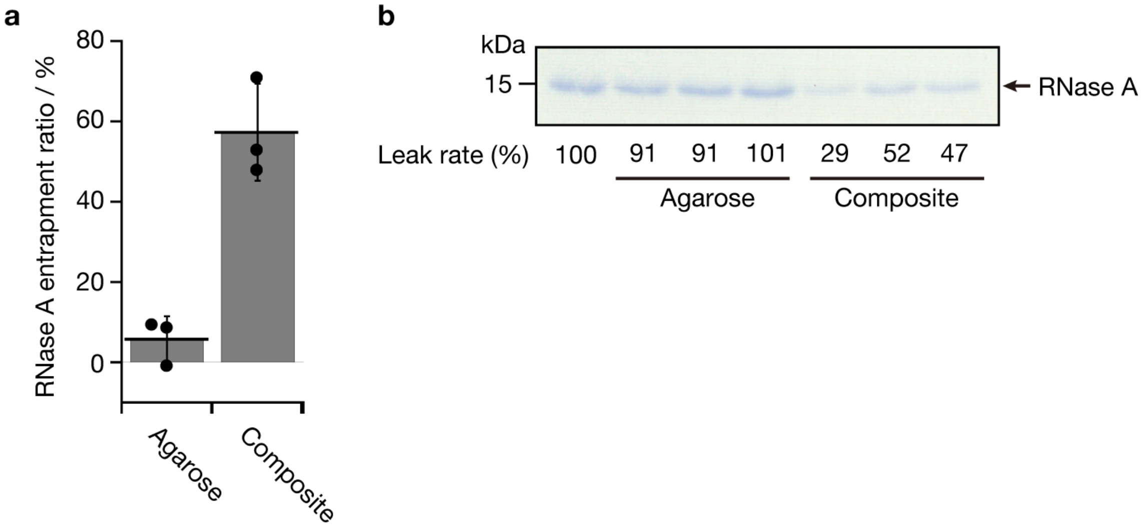

Supplementary Fig. 36. (**a**) RNase A entrapment ratio of the agarose and composite hydrogels (n = 3, the data represent the mean ± standard deviation). (**b**) SDS-PAGE analysis of RNase A entrapment in the agarose and composite hydrogels. The leak rates were calculated from the standard for 100% release. Condition: [**APmoc-F(CF$_3$)F**] = 0.6 wt%, [agarose] = 0.5 wt%, [RNase A] = 0.25 mg/mL, 100 mM HEPES, pH 8.0, 3 h.

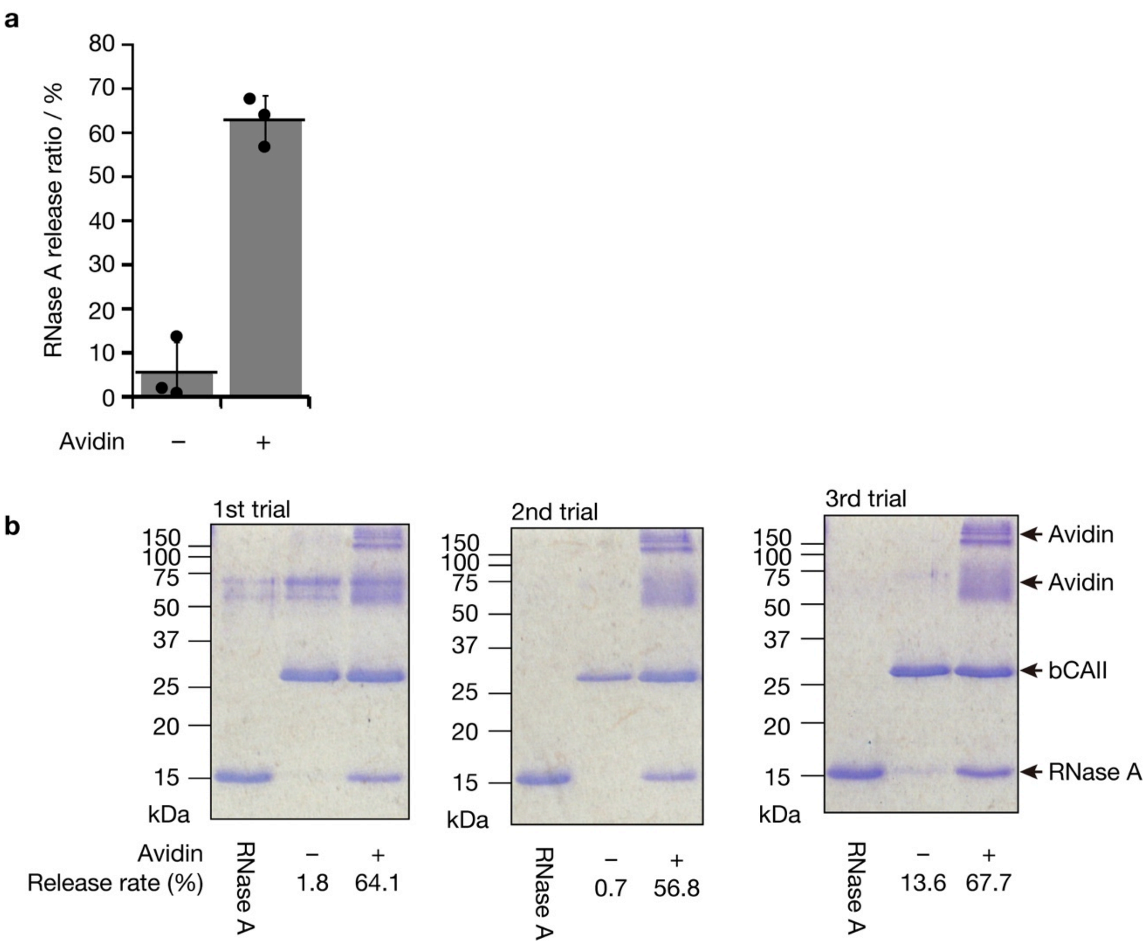

Supplementary Fig. 37. (**a**) Avidin-responsive RNase A release ratio from the composite hydrogel containing bCAII/**EAT(avidin)**. (**b**) SDS-PAGE analysis of avidin-responsive RNase A release from the composite hydrogel containing bCAII/**EAT(avidin)**. The lane of RNase A shows the standard for 100% release. Condition: [**APmoc-F(CF$_3$)F**] = 0.6 wt%, [agarose] = 0.5 wt%, [bCAII] = 10 μM, [**EAT(avidin)**] = 20 μM, [avidin] = 20 μM, [RNase A] = 0.25 mg/mL, 100 mM HEPES, pH 8.0, 3 h.

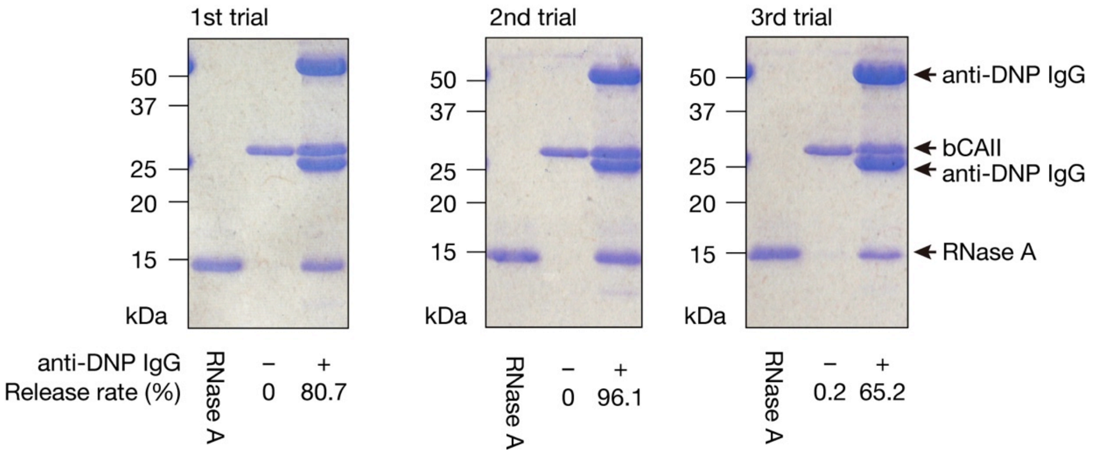

Supplementary Fig. 38. SDS-PAGE analysis of anti-DNP IgG-responsive RNase A release from the composite hydrogel containing bCAII/**EAT(DNP IgG)**. The lane of RNase A shows the standard for 100% release. Condition: [**APmoc-F(CF_3)F**] = 0.6 wt%, [agarose] = 0.5 wt%, [bCAII] = 10 μM, [**EAT(DNP IgG)**] = 15 μM, [anti-DNP IgG] = 15 μM, [RNase A] = 0.25 mg/mL, 100 mM HEPES, pH 8.0, 3 h.

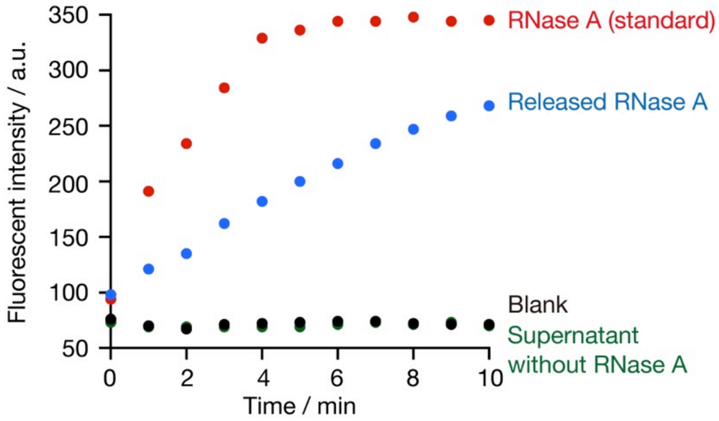

Supplementary Fig. 39. Determination of the enzymatic activity of RNase A. Red: RNase A (standard), blue: the released RNase A from the composite hydrogel, green: the supernatant without RNase A, black: blank buffer.

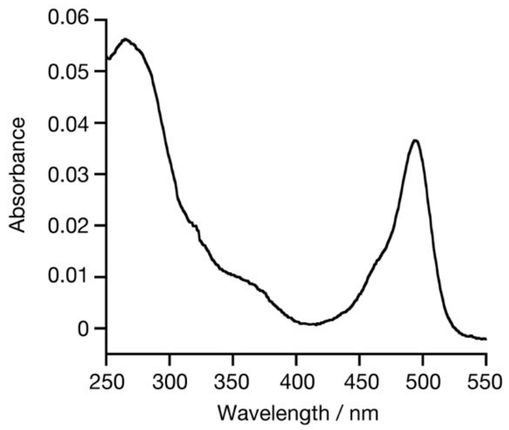

Supplementary Fig. 40. UV-vis absorption spectrum of FL-agarose. Condition: [FL-agarose] = 0.1 wt%, 10 mM tetraborate buffer, 25 ºC, optical length: 10 mm. The concentration of repeat units and fluorescein were 3.26 mM and 0.487 μM. Therefore, the modification ratio was determined to be 0.015 mol%.

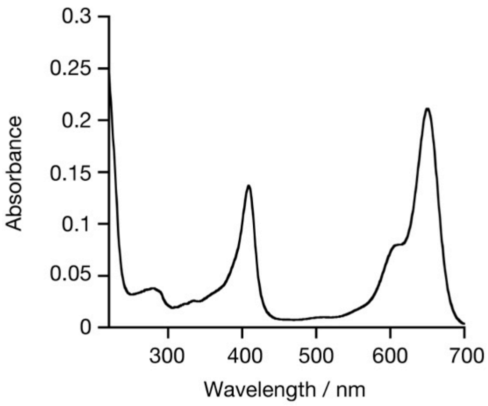

Supplementary Fig. 41. UV-vis absorption spectrum of Ax647-Mb. Concentrations of myoglobin and Ax647 were detemined to be 0.75 and 0.78 μM, respectively. Condition: PBS, 25 ºC, optical length: 10 mm.

Supplementary references

1. Wang, C.-W. *et al.* Steric-dependent label-free and washing-free enzyme amplified protein detection with dual-functional synthetic probes. *Anal. Chem.* **87**, 4231–4236 (2015).
2. Takaoka, Y. *et al.* Systematic study of protein detection mechanism of self-assembling ^{19}F NMR/MRI nanoprobes toward rational design and improved sensitivity. *J. Am. Chem. Soc.* **133**, 11725–11731 (2011).
3. Schindelin, J. *et al.* Fiji: an open-source platform for biological-image analysis. *Nat. Meth.* **9**, 676-682 (2012).
4. The molecular probes® handbook: a guide, 11th edition, Life Technologies Corporation (2010).
5. Castro-Forero, A., Jiménez, D., López-Garriga, J., Torres-Lugo, M. Immobilization of myoglobin from horse skeletal muscle in hydrophilic polymer networks. *J. Appl. Polym. Sci. Symp.* **15**, 107, 881–890 (2008).
