# Supplementary material for: Protein-responsive protein release of supramolecular/polymer hydrogel composite integrating enzyme activation systems
Source: Nat Commun. 2020 Jul 31;11:3859. doi: 10.1038/s41467-020-17698-0 (PMC7395795; doi:10.1038/s41467-020-17698-0)
Supplement: Supplementary file 3 — Description of Additional Supplementary Files [file 41467_2020_17698_MOESM3_ESM.docx]

Description of Additional Supplementary Files

Title: Supplementary movie 1

Description: Behaviors of APmoc-F(CF3)F and composite hydrogels when picked up with tweezers. Condition: [APmoc-F(CF3)F] = 0.6 wt%, [agarose] = 0 or 0.5 wt%, 100 mM HEPES, pH 8.0, rt.

Title: Supplementary movie 2

Description: The z-stack Airyscan CLSM images of the composite hydrogel (FL-agarose/APmoc-F(CF3)F) stained with TMR-Gua (the same as Fig. 4c). The image was rotated along x axis. Condition: [APmoc-F(CF3)F] = 0.6 wt%, [FL-agarose] = 0.5 wt%, [TMR-Gua] = 10 µM, 100 mM HEPES, pH 8.0.
